# Comparison of Remimazolam and Propofol for Sedation in Endoscopic Retrograde Cholangiopancreatography: A Systematic Review and Meta‐Analysis With Trial Sequential Analysis

**DOI:** 10.1111/den.15078

**Published:** 2025-07-02

**Authors:** In Jung Kim, Geun Joo Choi, Hyoung‐Chul Oh, Hyun Kang

**Affiliations:** ^1^ Department of Anesthesiology and Pain Medicine Chung‐Ang University College of Medicine Seoul Republic of Korea; ^2^ Department of Anesthesiology and Pain Medicine Chung‐Ang University Hospital Seoul Republic of Korea; ^3^ Division of Gastroenterology Chung‐Ang University College of Medicine Seoul Republic of Korea

**Keywords:** cholangiopancreatography, endoscopic retrograde, hypnotics and sedatives, propofol, remimazolam, sedation

## Abstract

**Objectives:**

Sedation for endoscopic retrograde cholangiopancreatography (ERCP) is challenging owing to patient comorbidities and procedural complexity. Remimazolam, a novel benzodiazepine, has potential safety benefits. We aimed to systematically compare the efficacy and safety of remimazolam and propofol for ERCP sedation through a meta‐analysis and trial sequential analysis (TSA).

**Methods:**

We searched Ovid‐MEDLINE, Ovid‐Embase, Cochrane Central, and Google Scholar for randomized controlled trials (RCTs) that compared efficacy and safety of remimazolam and propofol in ERCP sedation. Sensitivity analysis and TSA were also performed.

**Results:**

Five RCTs (965 participants) were included. In these trials, remimazolam significantly reduced hypoxia (risk ratio [RR], 0.522; 95% confidence interval [CI] 0.348–0.783; Grading of Recommendations, Assessment, Development, and Evaluation [GRADE], high), hypotension (RR, 0.507; 95% CI 0.396–0.649; GRADE, high), and bradycardia (RR, 0.475; 95% CI 0.308–0.732; GRADE, high). However, it increased tachycardia (RR, 3.363; 95% CI, 1.466–7.714; GRADE, moderate) and body movement (RR, 2.744; 95% CI, 1.216–6.193; GRADE, moderate). Delirium and agitation (RR, 0.586; 95% CI, 0.157–2.179; GRADE, moderate) and completion rate (RR, 1.009; 95% CI, 0.97–1.042; GRADE, moderate) were comparable. Recovery quality was higher in remimazolam group (mean difference, 1.541; 95% CI, 0.057–3.024; GRADE, low). Other outcomes, including induction and recovery times, were similar.

**Conclusion:**

Remimazolam demonstrated superior safety profile than propofol for ERCP sedation, significantly reducing hypoxia, hypotension, and bradycardia with high certainty evidence and TSA confirmation. Despite the higher incidence of tachycardia and body movement associated with remimazolam, the completion rate and risk of delirium or agitation were similar for both sedatives.

## Graphical abstract

This systematic review and meta‐analysis of 5 randomized controlled trials (*n* = 965) compares the safety profile of remimazolam versus propofol for sedation during ERCP. Remimazolam was associated with a significantly lower incidence of hypoxia, hypotension, and bradycardia. The findings support remimazolam as a safer alternative to propofol with high‐certainty evidence.

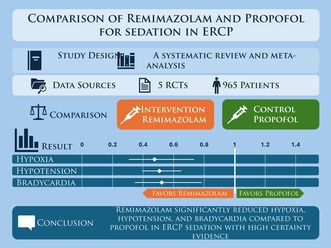

## 1 Introduction

Endoscopic retrograde cholangiopancreatography (ERCP) is one of the most technically challenging gastrointestinal endoscopic procedures, and managing sedation during this procedure is significantly difficult [1]. During ERCP, a relatively thick and rigid endoscope is used, which can cause substantial discomfort and physical irritation. Moreover, complex technical steps are involved in this procedure, as well as the risk of severe complications, such as acute pancreatitis, perforation, and bleeding [2, 3]. Thus, moderate‐to‐deep sedation is often necessary to ensure safe and effective ERCP.

However, achieving the appropriate level of sedation during ERCP remains challenging. Most patients who undergo ERCP are older population who frequently present with cardiovascular or respiratory comorbidities. Furthermore, ERCP is often required for conditions such as bile duct stones or malignant biliary obstruction, many of which require urgent intervention. In such emergencies, patients are often hemodynamically unstable, making sedation even more challenging [2, 4].

Propofol is widely used as the primary sedative agent in ERCP. Current guidelines for sedation in endoscopy recommend propofol‐based sedation to improve physician/patient satisfaction, procedural efficacy and completion, and recovery time [5, 6, 7]. It is effective in achieving moderate‐to‐deep sedation; however, its narrow therapeutic safety margin is a major concern because it is associated with an increased risk of hemodynamic instability [8, 9, 10, 11]. To overcome these limitations, combinations of propofol with other sedatives or analgesic agents have been explored in various studies [12, 13, 14]. Nonetheless, sedation during ERCP is demanding because it requires maintaining stable sedation to facilitate a technically complex procedure while simultaneously minimizing the risk of sedation‐related complications in vulnerable patients.

Remimazolam is a novel ultrashort‐acting benzodiazepine that acts on gamma‐aminobutyric acid (GABA) receptors [15, 16]. Recent studies on procedural sedation have reported that remimazolam is associated with a lower incidence of hypoxia and hypotension than propofol, suggesting a potential safety benefit with its use [17]. However, to date, the use of remimazolam and propofol for sedation during ERCP has not been specifically compared in systematic reviews or meta‐analyses.

It is crucial to systematically analyze and integrate the existing evidence on sedation strategies specific to ERCP. In this systematic review and meta‐analysis, we aimed to compare the efficacy and safety profiles of remimazolam and propofol for sedation during ERCP. Additionally, we conducted a trial sequential analysis (TSA) to assess the robustness of the available evidence and provide high‐quality methodological insights on the development of reliable sedation strategies for ERCP.

## 2 Methods

### 2.1 Protocol Registration and Reporting Guidelines

The protocol for the systematic review and meta‐analysis was developed following the Preferred Reporting Items for Systematic Review and Meta‐Analysis Protocols (PRISMA‐P) guidelines [18]. The protocol was registered in the International Prospective Register of Systematic Reviews (PROSPERO) (registration number: CRD42025384257) on January 2, 2025. The analysis was performed following the methodological recommendations of the Cochrane Collaboration [19, 20] and was reported in accordance with the Preferred Reporting Items for Systematic Reviews and Meta‐Analyses (PRISMA) statement [21].

### 2.2 Inclusion and Exclusion Criteria

Predefined inclusion and exclusion criteria were used to guide the systematic search. Studies that were eligible were full‐text reports of randomized controlled trials (RCTs) in which the efficacy and safety of remimazolam and propofol for sedation in patients undergoing ERCP were compared.

The PICO‐SD information was as follows:

- Patients (P): Adult participants undergoing ERCP sedation.
- Intervention (I): intravenous remimazolam administration with or without opioids for sedation.
- Comparison (C): intravenous propofol administration with or without opioids for sedation.
- Outcome measurements (O):

– The primary outcomes were the incidence of hypoxia, hypotension, hypertension, bradycardia, and tachycardia and time‐related outcomes of sedation, procedure, and recovery.
– Secondary outcomes: mean arterial pressure (MAP), heart rate (HR), completion rate, body movement, bispectral index (BIS), delirium or agitation, and outcomes reported on sedation, procedure, and recovery

- Study design (SD): Only full reports of RCTs were included.

The following were excluded: observational studies, conference abstracts, posters, case reports, case series, comments, letters to the editor, reviews, and laboratory or animal studies.

### 2.3 Information Source and Search Strategy

A comprehensive literature search was performed in February 2025 across the Ovid‐MEDLINE, Ovid‐Embase, Cochrane Central Register of Controlled Trials (CENTRAL), Web of Science, and Google Scholar databases. Two independent investigators (GJC and HCO) systematically searched for RCTs, in which the efficacy and safety of remimazolam and propofol in ERCP sedation were compared. The search strategy and search term are described in Data S1.

### 2.4 Study Selection and Data Extraction

The titles and abstracts of the reports identified via the search strategies were independently scanned by two independent investigators (GJC and HCO). The full paper was assessed separately by the two investigators for study inclusion.

Two independent investigators (IJK and GJC) systematically extracted relevant data from the included studies. Detailed study selection and data extraction process is described in Data S2.

### 2.5 Data Analysis

#### 2.5.1 Conventional Meta‐Analysis

All conventional meta‐analyses were performed using the Comprehensive Meta‐Analysis software (version 2.0; Englewood, NJ, USA, 2008). Pooled risk ratios (RRs) or mean difference (MD), and 95% confidence intervals (CIs) for each outcome were calculated.

To evaluate heterogeneity, we used Cochran's Q test, Higgins' I^2^, τ using the DerSimonian–Laird estimator, and the prediction interval (PI) methods to assess heterogeneity [22]. Heterogeneity was considered substantial if Cochran's Q test yielded a *p*‐value < 0.1 or if the I^2^ value exceeded 50% [23].

For heterogeneous outcomes, sensitivity analysis was conducted by removing one study at a time to identify its impact on the overall results. To estimate the clinical impact of the intervention, the number needed to treat (NNT) and 95% CI were calculated. Publication bias was not assessed because fewer than 10 studies were included [24]. For outcomes measured at multiple time points, we combined data across all time points for analysis.

#### 2.5.2 Trial Sequential Analysis

Conventional meta‐analyses are prone to random errors, especially when based on limited or sparse data. To address this, trial sequential analysis (TSA) was conducted [25]. TSA estimates the required information size (RIS)—the number of participants or events needed to detect a meaningful effect with sufficient power—and applies adjusted significance thresholds to reduce the risk of type I and II errors. In this study, TSA was used to assess whether the available evidence was sufficient and to calculate the RIS for each outcome. A random effects model generated cumulative Z‐curves based on the data from included studies.

To control the overall type I error rate at 5%, O'Brien–Fleming‐type monitoring boundaries were constructed to define thresholds for statistical significance (benefit or harm) and futility. If the Z‐curve crossed one of these boundaries, the evidence was considered strong enough to support or reject the treatment effect. If the curve stayed within the boundaries and the RIS had not been reached, the evidence was considered inconclusive.

A detailed description of conventional meta‐analysis and TSA is provided in Data S3.

### 2.6 Risk of Bias and Certainty of Evidence

Two investigators independently assessed the risk of bias for each included study using the Revised Cochrane Risk of Bias Tool for Randomized Trials (RoB 2.0) [26]. The GRADE system guideline was used to evaluate the certainty of evidence [27]. A detailed description of the risk of bias and certainty of evidence is provided in Data S4.

## 3 Results

### 3.1 Literature Search and Study Selection

In total, 537 records published until February 18, 2025 were retrieved from the Ovid‐MEDLINE, Ovid‐EMBASE, CENTRAL, and Google Scholar databases. Additionally, five records were identified through manual searches of clinical trial registries. After removing three duplicate articles, 539 records remained. Of these, 528 were excluded because the titles and abstracts of these studies were unsuitable. Specifically, exclusions were made for reviews, case reports, editorials and letters, study protocols, animal studies, nonrandomized controlled trials, studies not involving ERCP, pediatric studies, those with control groups other than propofol, and those conducted under general anesthesia. As a result, 11 articles remained for full‐text review. The interinvestigator agreement for this screening process was high (κ = 0.876).

**FIGURE 1:**
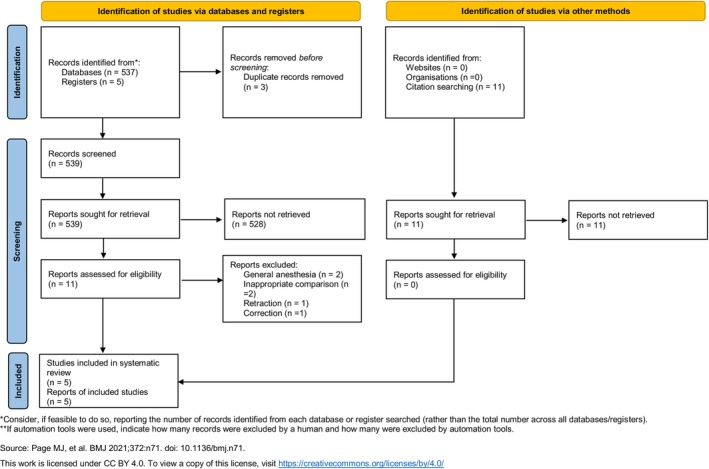
PRISMA flow diagram of the search for randomized controlled trials and the inclusion and exclusion criteria.

During the detailed full‐text evaluation of the remaining 11 articles, six studies were excluded for reasons such as ERCP performed under general anesthesia [3, 28], inappropriate comparisons [29, 30], retraction [31], and correction [32]. The investigators strongly agreed at this stage (κ = 0.820). Eleven records were obtained by manually searching OpenSIGLE and reference lists; however, these records were not further considered because they had already been captured in previous searches. Ultimately, five studies comprising 965 patients were included in the systematic review and meta‐analysis with TSA (Figure 1).

### 3.2 Study Characteristics

We included 965 patients from five trials [28, 33, 34, 35, 36, 37]. Of these, 479 (49.6%) received remimazolam, and 486 (50.4%) received propofol. In four of the five studies, opioids were administered: in two, alfentanil was used for both induction and maintenance [33, 35]; in one, butorphanol tartrate was used for induction with continuous infusion of remifentanil for maintenance [36]; in the final study, sufentanil was used for induction with continuous remifentanil infusion for maintenance [37]. Additionally, one study primarily focused on patients aged 75 years and older, which included 108 patients (10.3%) [35]. The detailed baseline characteristics of the patient populations and the induction, maintenance and rescue doses of remimazolam, and propofol in the included studies are shown in Table 1 and Table S1.

**TABLE 1:** Study characteristics of included studies (Remimazolam/Propofol).

Source	Age (years)	Sex (M/F) (n)	Weight (kg)	Height (cm)	BMI (kg/m^2^)	ASA (I/II/III)	Preop QOR‐15	Operation time (min)	Duration of anesthesia (min)
Dong, 2023	67 (58–74)/68 (58–75)	(144/106)/(156/99)	67.14 ± 9.51/66.16 ± 9.69	166.97 ± 7.89/167.02 ± 7.40	24.00 ± 2.28/23.64 ± 2.44	(87/123/40)/(82/132/41)	146 (143–147.25)/146 (143–148)	30 (24.5–42)/30 (21–37)	35 (27.75–45)/33 (24–40)
Lee, 2023	69 [38–88]/70 [27–92]	(32/23)/(32/23)	NR	NR	23.0 (14.9–32.7)/23.3 (17.3–43.3)	(10/36/9)/(11/37/7)	NR	979.2 ± 433.1/988.4 ± 464.0^b^	NR
Xin, 2024	81.5 ± 4.9/82.3 ± 6.0	(33/20)/(26/29)	NR	NR	22.3 ± 3.7/21.9 ± 3.5	(0/4/49)/(0/6/49)	134.7 ± 4.2/133.4 ± 4.5	NR	NR
Zhou, 2024	61.24 ± 12.15/59.98 ± 12.36	(23/28)/(26/25)	59.95 ± 11.86/59.14 ± 9.81	NR	22.06 ± 3.03/21.73 ± 2.68	(25/26)/(27/24)^a^	NR	75.45 ± 34.06/76.98 ± 33.32	82.76 ± 34.71/79.16 ± 27.31
Tian, 2024	78.11 ± 4.00/78 ± 3.63	(40/30)/(39/31)	NR	NR	23.1 ± 2.74/23 ± 2.74	(19/51/0)/(19/51/0)	NR	36.54 ± 6.22/36.8 ± 5.58	NR

*Note:* Values are expressed absolute number, mean ± standard deviation, median (Q_1_–Q_3_) or median [range].

Abbreviations: ASA‐PS, American Society of Anesthesiologists Physical Status; BMI, body mass index; F, female; M, male; n, number; NR, not reported; Preop‐QOR‐15, preoperative 15‐item questionnaire on quality of recovery.

^a^ I–II/III.

^b^ Expressed as second.

### 3.3 Respiratory and Hemodynamic Variables

#### 3.3.1 Hypoxia

**FIGURE 2:**
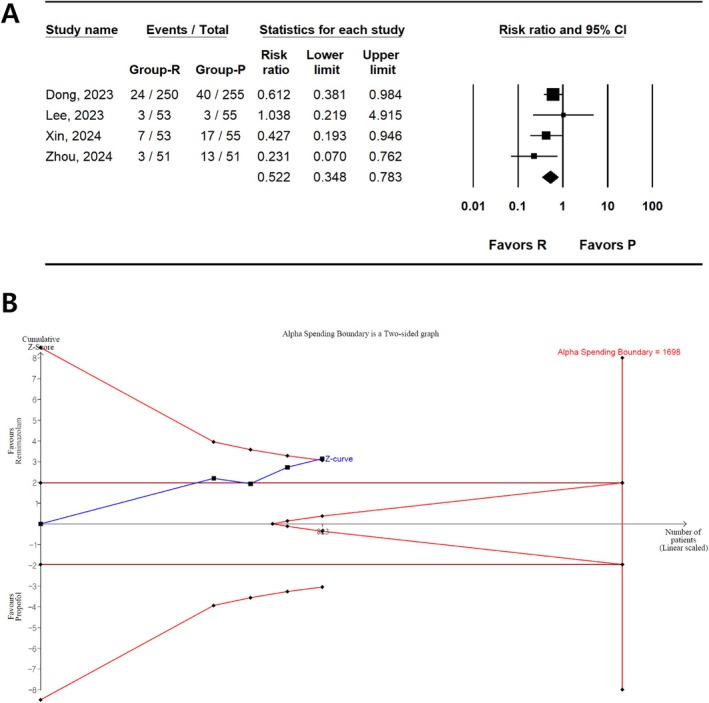
Incidence of hypoxia in remimazolam and propofol groups. (A) Forest plot. The figure depicts individual trials as filled squares, with a solid line representing the relative sample size and 95% confidence interval (CI) of the difference. The diamond shape represents the pooled estimate and uncertainty for the combined effect. The pooled estimate indicates that the incidence of hypoxia in the remimazolam group is lower than that in the propofol group. (B) Trial sequential analysis plot. The uppermost and lowest complete red curves represent the trial sequential monitoring boundary lines for benefit and harm, respectively. The horizontal dotted red line represents the conventional boundaries for statistical significance. The triangular red lines on the right side represent the futility boundaries. The blue solid line represents the cumulative Z‐curve. The number on the x‐axis indicates the required information size (*n* = 1698). The cumulative Z‐curve exceeds the conventional and trial sequential monitoring boundaries, favoring the use of remimazolam over propofol for the prevention of hypoxia.

In four studies involving 823 patients [33, 34, 35, 36], the incidence of hypoxia was reported. From the meta‐analysis results, a significantly lower risk of hypoxia was observed in the remimazolam group than in the propofol group (RR, 0.522; 95% CI 0.348–0.783; *I* ^2^ = 6.67; *P*_chi_ [2] = 0.360, τ = 0.119; 95% PI, 0.242–0.802; number needed to treat for benefit (NNTB), 12; 95% CI, NNTB 8–NNTB 26) (Figure 2A and Table 2). TSA revealed that 48.5% (823 of 1698 patients) of RIS was accrued. The cumulative Z‐curve crossed both the conventional test and trial sequential monitoring boundaries (Figure 2B).

**TABLE 2:** The summary of meta‐analysis and trial sequential analysis.

	No. of studies	No. of patients	Conventional meta‐analysis		Trial sequential analysis				NNT
			RR or MD with 95% CI	Heterogeneity (*I* ^2^, *P*_chi_^2^, τ, 95% PI)	Conventional test boundary	Monitoring boundary	Futility boundary	RIS	
Hypoxia	4	823	Significant (RR, 0.522; 95% CI 0.348–0.783)	*I* ^2^ = 6.67; *P*_chi_^2^ = 0.360, τ = 0.119; 95% PI, 0.242–0.802	Cross	Cross	Not cross	48.5% (823 of 1698 patients)	Significant (NNTB, 12; 95% CI, NNTB 8–NNTB 26)
Hypotension	4	823	Significant (RR, 0.507; 95% CI 0.396–0.649)	*I* ^2^ = 0.00; *P*_chi_^2^ = 0.657, τ = 0.00	Cross	Cross	Not cross	96.4% (613 of 636 patients)	Significant (NNTB, 6; 95% CI, NNTB 4–NNTB 9)
Hypertension	2	613	Not significant (RR, 1.060; 95% CI 0.646–1.742)	*I* ^2^ = 0.00; *P*_chi_^2^ = 0.522, τ = 0.00	Not cross	Not cross	Not cross	20.1% (613 of 3043 patients)	Not significant (NNTH, 186; 95% CI, NNTH 19 to ∞ to NNTB 25)
Bradycardia	4	823	Significant (RR, 0.475; 95% CI 0.308–0.732)	*I* ^2^ = 0.00; *P*_chi_^2^ = 0.722, τ = 0.00	Cross	Cross	Not cross	43.7% (823 of 1882 patients)	Significant (NNTB, 14; 95% CI, NNTB 9–NNTB 31)
Tachycardia	2	613	Significant (RR, 3.363; 95% CI 1.466–7.714)	*I* ^2^ = 0.00; *P*_chi_^2^ = 0.908, τ = 0.00	Cross	Not cross	Not cross	4.8% (613 of 12,640 patients)	Significant (NNTH, 19; 95% CI, NNTH 52–NNTH 11)
Mean arterial pressure	3	753	Not significant (MD, 4.152; 95% CI, −0.201 to 8.506)	*I* ^2^ = 84.78; *P*_chi_^2^ = 0.001; τ = 3.516; 95% PI, −10.335 to 18.639	NA	NA	NA	NA	NA
Heart rate	3	753	Significant (MD, 3.671; 95% CI, 2.121–5.220)	*I* ^2^ = 0.00; *P*_chi_^2^ = 0.817; τ = 0.000	NA	NA	NA	NA	NA
Completion rate	2	210	Not significant (RR, 1.010; 95% CI 0.976–1.044)	*I* ^2^ = 5.76; *P*_chi_^2^ = 0.303, τ = 0.00	Not cross	Not cross	Not cross	31.9% (210 of 659 patients)	NNTB, 53; 95% CI, NNTH 142 to ∞ to NNTB 22
Procedure time	2	613	Not significant (MD, −1.441; 95% CI, −4.516 to 1.635)	*I* ^2^ = 66.70; *P*_chi_^2^ = 0.083; τ = 1.819; 95% PI, −44.2 to 41.34	Not cross	Not cross	Cross	68.0% (613 of 902 patients)	NA
Body movement	2	210	Significant (RR, 2.744; 95% CI 1.216–6.193)	*I* ^2^ = 0.00; *P*_chi_^2^ = 0.704, τ = 0.00	Cross	Not cross	Not cross	5.0% (210 of 4174 patients)	Significant (NNTH, 9; 95% CI, NNTH 5–NNTH 35)
BIS	3	350	Significant (MD, 5.028; 95% CI, 0.937–9.749)	*I* ^2^ = 95.15; *P*_chi_^2^ < 0.001; τ = 3.674; 95% PI, −10.60 to 21.02	NA	NA	NA	NA	NA
Induction time	4	855	Not significant (MD, 3.922; 95% CI, −0.440 to 8.284)	*I* ^2^ = 89.14; *P*_chi_^2^ < 0.001; τ = 3.459; 95% PI, −7.08 to 14.93	Not cross	Not cross	Not cross	14.0% (855 of 6120 patients)	NA
Awake time	3	753	Not significant (MD, −0.531; 95% CI, −5.220 to 4.158)	*I* ^2^ = 99.42; *P*_chi_^2^ < 0.001; τ = 4.124; 95% PI, −18.28 to 17.21	Not cross	Not cross	Not cross	1.3% (753 of 57,688 patients)	NA
Recovery time	4	855	Not significant (MD, −0.237; 95% CI, −4.749 to 4.274)	*I* ^2^ = 99.36; *P*_chi_^2^ < 0.001; τ = 4.427; 95% PI, −10.95 to 10.48	Not cross	Not cross	Not cross	1.5% (855 of 57,892 patients)	NA
PACU time	3	350	Not significant (MD, −1.701; 95% CI, −5.738 to 2.337)	*I* ^2^ = 95.17; *P*_chi_^2^ < 0.001; τ = 3.467; 95% PI, −16.62 to 13.22	Not cross	Not cross	Not cross	4.56% (350 of 7679 patients)	NA
Injection Pain	3	715	Significant (RR, 0.054; 95% CI 0.009–0.339)	*I* ^2^ = 60.08; *P*_chi_^2^ = 0.082, τ = 1.251; 95% PI, 0.001–2.083	Cross	Not cross	Not cross	21.0% (715 of 3410 patients)	Significant (NNTB, 4; 95% CI, NNTB 3–NNTB 5)
Agitation and delirium	3	715	Not significant (RR, 0.586; 95% CI 0.157–2.179)	*I* ^2^ = 31.35; *P*_chi_^2^ = 0.233, τ = 0.66; 95% PI, 0.341–2.280	Not cross	Not cross	Not cross	2.9% (715 of 24,612 patients)	Not significant (NNTB, 61; 95% CI, NNTH 127 to ∞ to NNTB 25)
PONV	3	715	Not significant (RR, 1.032; 95% CI 0.863–1.234)	*I* ^2^ = 0.00; *P*_chi_^2^ = 0.738, τ = 0.00	Not cross	Not cross	Cross	Exceeds RIS (715 of 579 patients)	Not significant (NNTB, 101; 95% CI, NNTH 17 to ∞ to NNTB 12)
POD1_QOR15	2	613	Significant (MD, 1.541; 95% CI, 0.057–3.024)	*I* ^2^ = 53.16; *P*_chi_^2^ = 0.144; τ = 0.825; 95% PI, −8.94 to 12.02	Cross	Not cross	Not cross	59.1% (613 of 1038 patients)	NA

Abbreviations: BIS, Bispectral Index; CI, confidence interval; MD, mean difference; NA, not assessed; NNT, number needed to treat; NNTB, number needed to treat to benefit; NNTH, number needed to treat to harm; No, number; PACU, postanesthesia care unit; PI, predictive interval; POD1_QOR15, 15‐item questionnaire on quality of recovery at postoperative day 1; PONV; post‐operative nausea and vomiting; RIS, required information size; RR, relative risk.

#### 3.3.2 Hypotension

**FIGURE 3:**
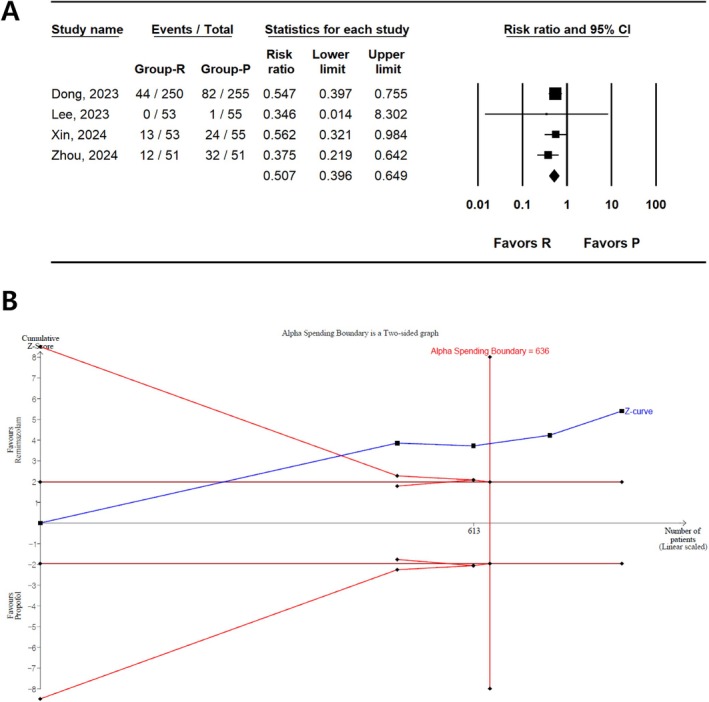
Incidence of hypotension in remimazolam and propofol groups. (A) Forest plot. The figure depicts individual trials as filled squares, with a solid line representing the relative sample size and 95% confidence interval (CI) of the difference. The diamond shape represents the pooled estimate and uncertainty for the combined effect. The pooled estimate indicates that the incidence of hypotension in remimazolam group is lower than that in the propofol group. (B) Trial sequential analysis plot. The uppermost and lowest complete red curves represent the trial sequential monitoring boundary lines for benefit and harm, respectively. The horizontal dotted red line represents the conventional boundaries for statistical significance. The triangular red lines on the right side represent the futility boundaries. The blue solid line represents the cumulative Z‐curve. The number on the x‐axis indicates the required information size (*n* = 636). The cumulative Z‐curve exceeds the conventional and trial sequential monitoring boundaries, favoring the use of remimazolam over propofol for the prevention of hypotension.

The incidence of hypotension was reported in four studies (823 patients) [33, 34, 35, 36]. The results showed a lower incidence of hypotension in the remimazolam group than in the propofol group (RR, 0.507; 95% CI 0.396–0.649; I^2^ = 0.00; *P*_chi_^2^ = 0.657, τ = 0.00; NNTB, 6; 95% CI, NNTB 4–NNTB 9) (Figure 3A, Table 2). TSA revealed that 96.4% (613 of 636 patients) of RIS were accrued. In addition, the cumulative Z‐curve crossed both the conventional test and trial sequential monitoring boundaries (Figure 3B).

#### 3.3.3 Hypertension

The incidence of hypertension was reported in two studies (613 patients) [33, 34]. The results showed no between‐group difference (RR, 1.060; 95% CI 0.646–1.742; *I* ^2^ = 0.00; *P*_chi_^2^ = 0.522, τ = 0.00; NNTB, 186; 95% CI, NNTH 19 to ∞ to NNTB 25) (Figure S1 and Table 2). TSA revealed that only 20.1% (613 of 3043 patients) of RIS were accrued. The cumulative Z‐curve did not cross the conventional test or trial sequential monitoring boundaries (Figure S2).

#### 3.3.4 Bradycardia

**FIGURE 4:**
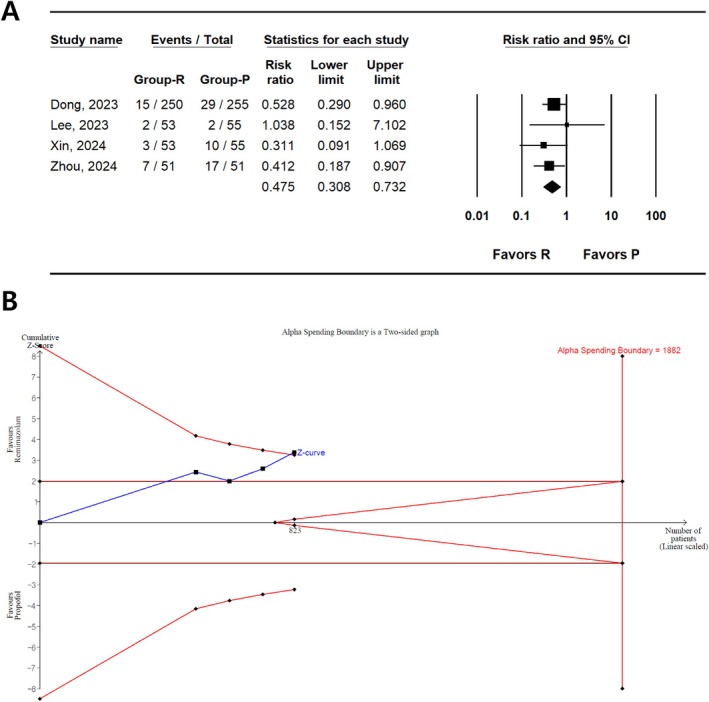
Incidence of bradycardia in remimazolam and propofol groups. (A) Forest plot. The figure depicts individual trials as filled squares, with a solid line representing the relative sample size and 95% confidence interval (CI) of the difference. The diamond shape represents the pooled estimate and uncertainty for the combined effect. The pooled estimate indicates that the incidence of bradycardia in remimazolam group is lower than that in the propofol group. (B) Trial sequential analysis plot. The uppermost and lowest complete red curves represent the trial sequential monitoring boundary lines for benefit and harm, respectively. The horizontal dotted red line represents the conventional boundaries for statistical significance. The triangular red lines on the right side represent the futility boundaries. The blue solid line represents the cumulative Z‐curve. The number on the x‐axis indicates the required information size (*n* = 1882). The cumulative Z‐curve exceeds the conventional and trial sequential monitoring boundaries, favoring the use of remimazolam over propofol for the prevention of bradycardia.

The incidence of bradycardia was reported in four studies (823 patients) [33, 34, 35, 36], showing a lower incidence of bradycardia in the remimazolam group than in the propofol group (RR, 0.475; 95% CI 0.308–0.732; *I* ^2^ = 0.00; *P*_chi_^2^ = 0.722, τ = 0.00; NNTB, 14; 95% CI, NNTB 9–NNTB 31) (Figure 4A and Table 2). TSA showed that 43.7% (823 of 1882 patients) of RIS were accrued. The cumulative Z‐curve also crossed both the conventional test and trial sequential monitoring boundaries (Figure 4B).

#### 3.3.5 Tachycardia

The incidence of tachycardia was reported in two studies (613 patients) [33, 34], showing a higher incidence of tachycardia in the remimazolam group than in the propofol group (RR, 3.363; 95% CI 1.466–7.714; I^2^ = 0.00; *P*_chi_^2^ = 0.908, τ = 0.00; NNTH, 19; 95% CI, NNTH 52–NNTH 11) (Figure S3 and Table 2). TSA showed that only 4.8% (613 of 12,640 patients) of RIS was accrued. The cumulative Z‐curve crossed the conventional test boundary but not the trial sequential monitoring boundary (Figure S4).

### 3.4 Mean Arterial Pressure (MAP) and Heart Rate

Mean arterial pressure was reported in three studies (753 patients) [33, 35, 37]. The findings revealed no between‐group difference (MD, 4.152; 95% CI, −0.201–8.506; *I* ^2^ = 84.78; *P*_chi_^2^ = 0.001; τ = 3.516; 95% PI, −10.335 to 18.639) (Figure S5 and Table 2). Additionally, heart rate was reported in three studies (753 patients) [33, 35, 37], showing higher heart rate in the remimazolam group than in the propofol group (MD, 3.671; 95% CI, 2.121–5.220; *I* ^2^ = 0.00; *P*_chi_^2^ = 0.817; τ = 0.000) (Figure S6). Because MAP and HR were measured at multiple time points, TSA was not performed.

### 3.5 Procedure Condition Variables

#### 3.5.1 Completion Rate

Completion rate was reported in two studies (210 patients) [34, 36], showing no between‐group difference (RR, 1.010; 95% CI 0.976–1.044; *I* ^2^ = 5.76; *P*_chi_^2^ = 0.303, τ = 0.00; NNTB, 53; 95% CI, NNTH 142 to ∞ to NNTB 22) (Figure S7 and Table 2). TSA showed that only 31.9% (210 of 659 patients) of RIS were accrued. The cumulative Z‐curve did not cross the conventional test or trial sequential monitoring boundaries (Figure S8).

#### 3.5.2 Body Movement

The incidence of body movement was reported in two studies (210 patients) [35, 36], showing higher incidence of body movement in the remimazolam group than in the propofol group (RR, 2.744; 95% CI 1.216–6.193; I^2^ = 0.00; *P*_chi_^2^ = 0.704, τ = 0.00; NNTH, 9; 95% CI, NNTH 5–NNTH 35) (Figure S9 and Table 2). TSA showed that only 5.0% (210 of 4174 patients) of RIS were accrued. The cumulative Z‐curve crossed the conventional test boundary but not the trial sequential monitoring boundary (Figure S10).

#### 3.5.3 Bispectral Index (BIS)

The BIS was reported in three studies (350 patients) [35, 36, 37], showing a higher BIS in the remimazolam group than in the propofol group (MD, 5.028; 95% CI, 0.937–9.749; *I* ^2^ = 95.15; *P*_chi_^2^ < 0.001; τ = 3.674; 95% PI, −10.60 to 21.02) (Figure S11 and Table 2). BIS was measured at multiple time points; hence, TSA analysis was not performed.

### 3.6 Other Variables

#### 3.6.1 Agitation or Delirium

The incidence of agitation or delirium was reported in three studies (715 patients) [33, 35, 36], showing no between‐group difference (RR, 0.586; 95% CI 0.157–2.179; *I* ^2^ = 31.35; *P*_chi_^2^ = 0.233, τ = 0.66; 95% PI, 0.341–2.280; NNTB, 61; 95% CI, NNTH 127 to ∞ to NNTB 25) (Figure S12 and Table 2). TSA revealed that only 2.9% (715 of 24,612 patients) of the RIS were accrued. The cumulative Z‐curve did not cross the conventional test or trial sequential monitoring boundaries (Figure S13).

#### 3.6.2 Quality of Recovery at Post‐ERCP Day 1

In two studies (613 patients), a 15‐item questionnaire was used to assess the quality of recovery at postoperative day 1 (POD1_QOR15) [33, 35]. The results showed a higher POD1_QOR15 in the remimazolam group than in the propofol group (MD, 1.541; 95% CI, 0.057–3.024; *I* ^2^ = 53.16; *P*_chi_^2^=0.144; τ = 0.825; 95% PI, −8.94 to 12.02) (Figure S14 and Table 2). TSA showed that only 59.1% (613 of 1038 patients) of the RIS was accrued. The cumulative Z‐curve crossed the conventional test boundary but did not cross the trial sequential monitoring boundary (Figure S15).

Other outcomes—including time‐related variables such as procedure time, induction time, time to awakening, recovery time, and PACU stay—were similar between the two groups. The incidence of postoperative nausea and vomiting (PONV) was also comparable. However, the incidence of injection pain was lower in the remimazolam group compared to the propofol group. Detailed data are presented in Table 2 and Figures S16–S29.

### 3.7 Risk of Bias

The risk‐of‐bias assessment using the Cochrane tool is presented in Table S2. In all the included studies, we found a low risk of bias concerning deviations from the intended intervention, missing outcome data, and selective reporting of results. However, in three studies [34, 35, 37], concerns were raised about the potential bias in the randomization process. Additionally, in three studies [34, 35, 37], the risk of bias in outcome measurement was considered a concern because of the lack of information on whether the outcome assessors were blinded. This absence of blinding could have introduced bias because the assessors' knowledge of the intervention may influence the outcome evaluation. For outcomes in which the assessment was unlikely to be affected by knowledge of the intervention, the risk of bias in the outcome measurement was judged to be low.

### 3.8 Quality of the Evidence

GRADE quality of evidence for each outcome is presented in Table S3. Nineteen outcomes were evaluated using the GRADE system. The quality of the pooled analysis of the outcomes of hypoxia, hypotension, and bradycardia was rated high. The quality was rated as moderate for hypertension, tachycardia, heart rate, completion rate, body movement, agitation, delirium, and postoperative nausea and vomiting. For the remaining outcomes, the quality of the pooled analysis was rated low.

## 4 Discussion

In this study, we found that remimazolam was associated with significantly fewer episodes of hypoxia, hypotension, and bradycardia than propofol during ERCP. The GRADE assessment of these outcomes was rated high, and through the TSA, we confirmed the robustness and conclusiveness of these outcomes as the cumulative Z‐curve crossed the trial sequential monitoring boundary. This suggests that the evidence is strong enough to support the findings, making further studies unnecessary, despite the limited number of included trials.

These findings are particularly significant considering that patients undergoing ERCP are highly susceptible to complications, such as hypoxia, hypotension, and bradycardia, which are major concerns when using propofol for sedation [38, 39, 40, 41]. During ERCP sedation, airway management becomes more challenging owing to transoral endoscope insertion [42, 43], and prone or lateral positioning of these patients further elevates the risk of hypoxia and hypotension [44, 45]. The results of this study align with those of a previous study in which remimazolam was compared with propofol in gastrointestinal endoscopic procedures [46]. These findings support the use of evidence‐based sedation options for ERCP, particularly in patients at risk of cardiopulmonary complications.

Despite its benefits, remimazolam is associated with a higher incidence of tachycardia and body movements than propofol. Excessive movement during ERCP sedation can interfere with the procedure, suggesting that remimazolam may be less effective in maintaining moderate‐to‐deep sedation. Additionally, the higher BIS values observed in the remimazolam group indicated that the depth of sedation may not be consistently maintained.

Furthermore, the higher incidence of tachycardia in the remimazolam group may be associated with the increased body movements observed in this group. During ERCP, antispasmodic agents are often used. Propofol may help counteract the tachycardic effects of the antispasmodic agents; meanwhile, remimazolam does not appear to have these effects. This highlights the need for caution when using remimazolam, particularly in patients requiring strict heart rate control, such as those with atrial fibrillation or aortic stenosis. The procedure completion rates were comparable despite these concerns, and patients in the remimazolam group reported better recovery quality on the following day, suggesting that this potential disadvantage may not have significant clinical implications.

We found that the mean patient age exceeded 60 years in this study. However, remimazolam did not increase the incidence of delirium or agitation. Given the concerns regarding benzodiazepine use and delirium in older adults, similar risks might have been expected with remimazolam. Nonetheless, in a preceding meta‐analysis, there was no higher incidence of postoperative delirium with remimazolam than with propofol for procedural sedation [17]. Additionally, flumazenil, a reversal agent, enhances safety and helps manage agitation. However, caution should be taken when using remimazolam for patients who are resedated after general anesthesia [47].

The evaluated outcomes, including tachycardia, body movement, completion rate, and delirium or agitation, were rated as having moderate certainty based on GRADE. TSA showed that the Z‐curve did not cross the monitoring boundary, leaving the evidence inconclusive. Further studies with larger data sets are needed to refine ERCP sedation strategies. Furthermore, exploring adjunct sedatives or analgesics may help optimize remimazolam‐based sedation, and additional data on the incidence of delirium in older patients would be valuable.

In this study, trial sequential analysis (TSA) was used to reduce random errors and account for repeated significance testing. Each outcome was analyzed with a 5% type I error rate, but no adjustment was made for multiple outcomes, which may increase the risk of false positives.

As TSA was conducted retrospectively using existing data, the monitoring boundaries served as reference points rather than predefined stopping rules. In some cases (e.g., Figures S13, S21, S23, and S25), the cumulative Z‐curve crossed a boundary and later returned within it, possibly due to random variation, limited sample size, or updated effect estimates. These patterns suggest that early conclusions may be less stable. Retrospective TSA should be interpreted as exploratory and should be used to contextualize findings with uncertain or evolving evidence.

This study had some limitations. First, only five RCTs were included, reflecting the limited research on remimazolam use in ERCP possibly due to its recent introduction to the clinical field and the complexity of the procedure. However, TSA was used to minimize type I errors and assess result robustness. Second, all studies were conducted in East Asia (four in China, one in South Korea), potentially limiting generalizability to non‐Asian populations. Hence, further research is required to confirm these findings in diverse ethnic and geographical groups. Third, subgroup analyses for BMI, ASA classification, and opioid regimen were not conducted owing to insufficient data, although these factors are relevant to cardiopulmonary risk. Thus, the safety assessments should be improved in future studies incorporating these variables. Additionally, most patients were over 60 years old; nonetheless, more data on very old adults would further clarify the safety of remimazolam. Finally, considerable clinical heterogeneity was observed among the included studies, particularly regarding the type, use, and dosage of co‐administered opioids, as well as the administration methods and dosages of remimazolam and propofol. Although sensitivity analyses supported the robustness of the findings, the potential for residual confounding cannot be entirely ruled out. These factors should be considered when interpreting the results.

Despite these limitations, this study has several strengths. All the included studies were RCTs, which enhanced the validity of the results. A rigorous methodological approach was followed, which included preregistration and TSA, ensuring result robustness. The risk of bias was thoroughly assessed at the outcome level for each study, further reinforcing reliability. In addition, outcome heterogeneity was generally low, except for MAP and BIS values, which were possibly caused by differences in measurement time points. Notably, this is the first study in which remimazolam was directly compared with propofol for ERCP sedation, providing valuable insights into the strengths and weaknesses of both drugs and addressing a critical gap in the existing literature.

In conclusion, remimazolam had a superior safety profile compared with propofol, making it a promising option for ERCP sedation. The evidence that remimazolam reduces the incidence of hypoxia, hypotension, and bradycardia is supported with high certainty and TSA‐confirmed robustness. Although the outcomes of remimazolam were comparable in terms of procedural completion rate, delirium or agitation incidence, and time‐related factors such as induction and recovery time, remimazolam was associated with a higher occurrence of tachycardia and body movement. Moreover, it improved post‐ERCP recovery quality. Considering the risks associated with ERCP and patient conditions, evidence‐based sedation strategies should be carefully applied to enhance patient safety and optimize procedural outcomes.

## Author Contributions

I.J.K., G.J.C., and H.‐C.O. designed the search template and performed the search and data collection. Data analysis was performed by I.J.K. and H.‐C.O., bias assessment by I.J.K. and G.J.C., and quality of evidence by G.J.C. and H.K. All authors contributed to the manuscript development and participated in the critical scrutiny and revision of the manuscript. All the authors have read and approved the final version of the manuscript. The corresponding author attests that all listed authors meet the authorship criteria and that no others meeting the criteria have been omitted.

## Ethics Statement

The authors have nothing to report.

## Consent

The authors have nothing to report.

## Conflicts of Interest

The authors declare no conflicts of interest.

## Supporting information


**Figure S1.** Forest plot for hypertension comparing remimazolam and propofol. The figure depicts individual trials as filled squares with relative sample size and the 95% confidence interval (CI) of the difference as a solid line. The diamond shape indicates the pooled estimate and uncertainty for the combined effect. The pooled estimate indicates no significant difference in the incidence of hypertension between remimazolam and propofol.
**Figure S2.** Trial sequential analysis plot for hypertension comparing remimazolam and propofol. Uppermost and lowermost complete red curves represent trial sequential monitoring boundary lines for benefit and harm, respectively. Horizontal dotted red line represents the conventional boundaries for statistical significance. Triangular red lines on the right side reflects the futility boundaries. The blue solid line represents the cumulative z‐curve. The number on the x‐axis indicates required information size (*n* = 3043). The TSA suggests insufficient evidence, with only 20.1% of the required information size (RIS) accrued, as the Z‐curve crossed neither the conventional test boundary nor cross the trial sequential monitoring boundary.
**Figure S3.** Forest plot for tachycardia comparing remimazolam and propofol. The figure depicts individual trials as filled squares with relative sample size and the 95% confidence interval (CI) of the difference as a solid line. The diamond shape indicates the pooled estimate and uncertainty for the combined effect. The pooled estimate indicates significant difference in the incidence of tachycardia between remimazolam and propofol.
**Figure S4.** Trial sequential analysis plot for tachycardia comparing remimazolam and propofol. Horizontal dotted red line represents the conventional boundaries for statistical significance. The blue solid line represents the cumulative z‐curve. The TSA suggests insufficient evidence, with only 4.8% (613 of 12,640 patients) of the required information size (RIS) accrued, as the Z curve crossed the conventional test boundary, but did not cross the trial sequential monitoring boundary.
**Figure S5.** Forest plot for MAP comparing remimazolam and propofol. The figure depicts individual trials as filled squares with relative sample size and the 95% confidence interval (CI) of the difference as a solid line. The diamond shape indicates the pooled estimate and uncertainty for the combined effect. The pooled estimate indicates no significant difference in intraoperative MAP between remimazolam and propofol.
**Figure S6.** Forest plot for HR comparing remimazolam and propofol. The figure depicts individual trials as filled squares with relative sample size and the 95% confidence interval (CI) of the difference as a solid line. The diamond shape indicates the pooled estimate and uncertainty for the combined effect. The pooled estimate indicates significant difference in intraoperative HR between remimazolam and propofol.
**Figure S7.** Forest plot for completion rate comparing remimazolam and propofol. The figure depicts individual trials as filled squares with relative sample size and the 95% confidence interval (CI) of the difference as a solid line. The diamond shape indicates the pooled estimate and uncertainty for the combined effect. The pooled estimate indicates no significant difference in completion rate between remimazolam and propofol.
**Figure S8.** Trial sequential analysis plot for completion rate comparing remimazolam and propofol. Uppermost and lowermost complete red curves represent trial sequential monitoring boundary lines for benefit and harm respectively. Horizontal dotted red line represents the conventional boundaries for statistical significance. Triangular red lines on the right side reflects the futility boundaries. The blue solid line represents the cumulative z‐curve. The number on the x‐axis indicates required information size (*n* = 659). The TSA suggests insufficient evidence, with only 31.9% of the required information size (RIS) accrued, as the Z‐curve crossed neither the conventional test boundary nor cross the trial sequential monitoring boundary.
**Figure S9.** Forest plot for body movement comparing remimazolam and propofol. The figure depicts individual trials as filled squares with relative sample size and the 95% confidence interval (CI) of the difference as a solid line. The diamond shape indicates the pooled estimate and uncertainty for the combined effect. The pooled estimate indicates significant difference in body movement between remimazolam and propofol.
**Figure S10.** Trial sequential analysis plot for body movement comparing remimazolam and propofol. Horizontal dotted red line represents the conventional boundaries for statistical significance. Triangular red lines on the right side reflects the futility boundaries. The blue solid line represents the cumulative z‐curve. The number on the x‐axis indicates required information size (*n* = 4174). The TSA suggests insufficient evidence, with only 5.0% of the required information size (RIS) accrued, as the Z‐curve crossed the conventional test boundary but did not cross the trial sequential monitoring boundary.
**Figure S11.** Forest plot for BIS comparing remimazolam and propofol. The figure depicts individual trials as filled squares with relative sample size and the 95% confidence interval (CI) of the difference as a solid line. The diamond shape indicates the pooled estimate and uncertainty for the combined effect. The pooled estimate indicates significant difference in BIS between remimazolam and propofol.
**Figure S12.** Forest plot for agitation and delirium comparing remimazolam and propofol. The figure depicts individual trials as filled squares with relative sample size and the 95% confidence interval (CI) of the difference as a solid line. The diamond shape indicates the pooled estimate and uncertainty for the combined effect. The pooled estimate indicates significant difference in injection pain between remimazolam and propofol.
**Figure S13.** Trial sequential analysis plot for agitation and delirium comparing remimazolam and propofol. Horizontal dotted red line represents the conventional boundaries for statistical significance. The blue solid line represents the cumulative z‐curve. The TSA suggests insufficient evidence, with only 2.9% (715 of 24,612 patients) of the required information size (RIS) accrued, as the Z‐curve crossed neither the conventional test boundary nor cross the trial sequential monitoring boundary.
**Figure S14.** Forest plot for POD1 QOR15 comparing remimazolam and propofol. The figure depicts individual trials as filled squares with relative sample size and the 95% confidence interval (CI) of the difference as a solid line. The diamond shape indicates the pooled estimate and uncertainty for the combined effect. The pooled estimate indicates no significant difference in PONV between remimazolam and propofol.
**Figure S15.** Trial sequential analysis plot for POD1 QOR15 comparing remimazolam and propofol. Uppermost and lowermost complete red curves represent trial sequential monitoring boundary lines for benefit and harm respectively. Horizontal dotted red line represents the conventional boundaries for statistical significance. Triangular red lines on the right side reflects the futility boundaries. The blue solid line represents the cumulative z‐curve. The number on the x‐axis indicates required information size (*n* = 1038). The TSA suggests insufficient evidence, with only 59.1% of the required information size (RIS) accrued, as the Z‐curve crossed the conventional test boundary but did not cross the trial sequential monitoring boundary.
**Figure S16.** Forest plot for procedure time comparing remimazolam and propofol. The figure depicts individual trials as filled squares with relative sample size and the 95% confidence interval (CI) of the difference as a solid line. The diamond shape indicates the pooled estimate and uncertainty for the combined effect. The pooled estimate indicates no significant difference in procedure time between remimazolam and propofol.
**Figure S17.** Trial sequential analysis plot for procedure time comparing remimazolam and propofol. Uppermost and lowermost complete red curves represent trial sequential monitoring boundary lines for benefit and harm, respectively. Horizontal dotted red line represents the conventional boundaries for statistical significance. Triangular red lines on the right side reflects the futility boundaries. The blue solid line represents the cumulative z‐curve. The number on the x‐axis indicates required information size (*n* = 902). The TSA suggests insufficient evidence, with only 68.0% of the required information size (RIS) accrued. However, the cumulative Z curve crossed the futility boundary, suggesting no further studies were needed.
**Figure S18.** Forest plot for induction time comparing remimazolam and propofol. The figure depicts individual trials as filled squares with relative sample size and the 95% confidence interval (CI) of the difference as a solid line. The diamond shape indicates the pooled estimate and uncertainty for the combined effect. The pooled estimate indicates no significant difference in induction time between remimazolam and propofol.
**Figure S19.** Trial sequential analysis plot for induction time comparing remimazolam and propofol. Uppermost and lowermost complete red curves represent trial sequential monitoring boundary lines for benefit and harm respectively. Horizontal dotted red line represents the conventional boundaries for statistical significance. Triangular red lines on the right side reflects the futility boundaries. The blue solid line represents the cumulative z‐curve. The number on the x‐axis indicates required information size (*n* = 6120). The TSA suggests insufficient evidence, with only 14.0% of the required information size (RIS) accrued, as the Z‐curve crossed neither the conventional test boundary nor cross the trial sequential monitoring boundary.
**Figure S20.** Forest plot for awake time comparing remimazolam and propofol. The figure depicts individual trials as filled squares with relative sample size and the 95% confidence interval (CI) of the difference as a solid line. The diamond shape indicates the pooled estimate and uncertainty for the combined effect. The pooled estimate indicates no significant difference in awake time between remimazolam and propofol.
**Figure S21.** Trial sequential analysis plot for awake time comparing remimazolam and propofol. Horizontal dotted red line represents the conventional boundaries for statistical significance. The blue solid line represents the cumulative z‐curve. The TSA suggests insufficient evidence, with only 1.3% (753 of 57,688 patients) of the required information size (RIS) accrued, as the Z‐curve crossed neither the conventional test boundary nor cross the trial sequential monitoring boundary.
**Figure S22.** Forest plot for recovery time comparing remimazolam and propofol. The figure depicts individual trials as filled squares with relative sample size and the 95% confidence interval (CI) of the difference as a solid line. The diamond shape indicates the pooled estimate and uncertainty for the combined effect. The pooled estimate indicates no significant difference in recovery time between remimazolam and propofol.
**Figure S23.** Trial sequential analysis plot for recovery time comparing remimazolam and propofol. Horizontal dotted red line represents the conventional boundaries for statistical significance. The blue solid line represents the cumulative z‐curve. The TSA suggests insufficient evidence, with only 1.5% (855 of 57,892 patients) of the required information size (RIS) accrued, as the Z‐curve crossed neither the conventional test boundary nor cross the trial sequential monitoring boundary.
**Figure S24.** Forest plot for PACU time comparing remimazolam and propofol. The figure depicts individual trials as filled squares with relative sample size and the 95% confidence interval (CI) of the difference as a solid line. The diamond shape indicates the pooled estimate and uncertainty for the combined effect. The pooled estimate indicates no significant difference in PACU time between remimazolam and propofol.
**Figure S25.** Trial sequential analysis plot for PACU time comparing remimazolam and propofol. Horizontal dotted red line represents the conventional boundaries for statistical significance. The blue solid line represents the cumulative Z‐curve. The TSA suggests insufficient evidence, with only 4.56% of the required information size (RIS) accrued, as the Z‐curve crossed neither the conventional test boundary nor cross the trial sequential monitoring boundary.
**Figure S26.** Forest plot for PONV comparing remimazolam and propofol. The figure depicts individual trials as filled squares with relative sample size and the 95% confidence interval (CI) of the difference as a solid line. The diamond shape indicates the pooled estimate and uncertainty for the combined effect. The pooled estimate indicates no significant difference in PONV between remimazolam and propofol.
**Figure S27.** Trial sequential analysis plot for PONV comparing remimazolam and propofol. Uppermost and lowermost complete red curves represent trial sequential monitoring boundary lines for benefit and harm respectively. Horizontal dotted red line represents the conventional boundaries for statistical significance. Triangular red lines on the right side reflects the futility boundaries. The blue solid line represents the cumulative z‐curve. The number on the x‐axis indicates required information size (*n* = 579). The results show that patients enrolled exceeds RIS (735 of 446 patients), yet the cumulative Z‐curve intersects futility boundary, suggesting no further studies were needed.
**Figure S28.** Forest plot for injection pain comparing remimazolam and propofol. The figure depicts individual trials as filled squares with relative sample size and the 95% confidence interval (CI) of the difference as a solid line. The diamond shape indicates the pooled estimate and uncertainty for the combined effect. The pooled estimate indicates significant difference in injection pain between remimazolam and propofol.
**Figure S29.** Trial sequential analysis plot for injection pain comparing remimazolam and propofol. Uppermost and lowermost complete red curves represent trial sequential monitoring boundary lines for benefit and harm, respectively. Horizontal dotted red line represents the conventional boundaries for statistical significance. Triangular red lines on the right side reflects the futility boundaries. The blue solid line represents the cumulative Z‐curve. The number on the x‐axis indicates required information size (*n* = 3410). The TSA suggests insufficient evidence, with only 21.0% of the required information size (RIS) accrued, as the Z‐curve crossed the conventional test boundary but did not cross the trial sequential monitoring boundary.
**Table S1.** The induction, maintenance, and rescue doses of remimazolam, propofol, and opioid.
**Table S2.** Risk of bias.
**Table S3.** The GRADE evidence quality for each outcome.

**Data S1.** Search terms for Ovid‐MEDLINE and Ovid‐Embase.
**Data S2.** Details of conventional meta‐analysis and trial sequential analysis.
**Data S3.** Methodological assessment of risk of bias and evidence certainty using RoB 2.0 and GRADE.

## Data Availability

All data generated or analyzed during this study are included in this published article (and its Data S1).
