# Supplementary material for: Comparison of Remimazolam and Propofol for Sedation in Endoscopic Retrograde Cholangiopancreatography: A Systematic Review and Meta‐Analysis With Trial Sequential Analysis
Source: Dig Endosc. 2025 Jul 2;37(10):1054–67. doi: 10.1111/den.15078 (PMC12511914; doi:10.1111/den.15078)
Supplement: Supplementary file 1 — Figure S1. Forest plot for hypertension comparing remimazolam and propofol. The figure depicts individual trials as filled squares with relative sample size and the 95% confidence interval (CI) of the difference as a solid line. The diamond shape indicates the pooled estimate and uncertainty for the combined effect. The pooled estimate indicates no significant difference in the incidence of hypertension between remimazolam and propofol. Figure S2. Trial sequential analysis plot for hypertension comparing remimazolam and propofol. Uppermost and lowermost complete red curves represent trial sequential monitoring boundary lines for benefit and harm, respectively. Horizontal dotted red line represents the conventional boundaries for statistical significance. Triangular red lines on the right side reflects the futility boundaries. The blue solid line represents the cumulative z‐curve. The number on the x‐axis indicates required information size (n = 3043). The TSA suggests insufficient evidence, with only 20.1% of the required information size (RIS) accrued, as the Z‐curve crossed neither the conventional test boundary nor cross the trial sequential monitoring boundary. Figure S3. Forest plot for tachycardia comparing remimazolam and propofol. The figure depicts individual trials as filled squares with relative sample size and the 95% confidence interval (CI) of the difference as a solid line. The diamond shape indicates the pooled estimate and uncertainty for the combined effect. The pooled estimate indicates significant difference in the incidence of tachycardia between remimazolam and propofol. Figure S4. Trial sequential analysis plot for tachycardia comparing remimazolam and propofol. Horizontal dotted red line represents the conventional boundaries for statistical significance. The blue solid line represents the cumulative z‐curve. The TSA suggests insufficient evidence, with only 4.8% (613 of 12,640 patients) of the required information size (RIS) accrued, as the [file DEN-37-1054-s002.docx]

**Supplementary Document 1.** Search terms for Ovid-MEDLINE and Ovid-Embase

**Search terms for Ovid-MEDLINE**

1. randomized controlled trial.pt
2. randomized controlled trial$.mp
3. controlled clinical trial.pt
4. controlled clinical trial$.mp
5. random allocation.mp
6. exp double-blind method/
7. double-blind.mp
8. exp single-blind method/
9. single-blind.mp
10. or/1-9
11. clinical trial.pt
12. clinical trial$.mp
13. exp clinical trial/
14. (clin$ adj25 trial$).mp
15. ((singl$ or doubl$ or tripl$ or trebl$) adj25 (blind$ or mask$)).mp
16. random$.mp
17. exp research design/
18. research design.mp
19. or/11-18
20. 10 or 19
21. Case report.tw.
22. Letter.pt.
23. Historical article.pt.
24. Review.pt.
25. or/21-24
26. 20 not 25
27. Exp remimazolam/
28. remimazolam.mp.
29. Byfavo.mp
30. or/27-29
31. 26 and 30

**Search terms for Ovid-Embase**

1. randomi?ed controlled trial$:ti,ab
2. 'controlled clinical trial (topic)'/exp
3. controlled AND clinical AND trials
4. controlled clinical trial$.mp.
5. 'randomization'/exp
6. 'random allocation'/exp
7. random AND allocation:ti,ab
8. ‘double blind’:ti,ab
9. ‘single blind’:ti,ab
10. #1 OR #2 OR #3 OR #4 OR #5 OR #6 OR #7 OR #8 OR #9
11. 'clinical trial (topic)'/exp
12. clinical AND trial$:ti,ab
13. random$:ti,ab
14. rct:ti,ab
15. #11 OR #12 OR #13 OR #14
16. #10 OR #15
17. 'case study'/exp
18. 'case report'/exp
19. 'abstract report'/exp
20. 'letter'/exp
21. #16 NOT #20
22. 'remimazolam'/exp
23. remimazolam:ti,ab
24. Byfavo:ti,ab
25. #23 OR #24 OR #25
26. #22 AND #26

**Supplementary document 2.** Details of conventional meta-analysis and trial sequential analysis

**Conventional Meta-analysis**

All conventional meta-analyses were performed using the Comprehensive Meta-Analysis software (version 2.0; Englewood, NJ, USA, 2008). Two independent investigators (IJK and GJC) inputted all data into the software and calculated the pooled risk ratios (RRs) or mean difference (MD), and 95% confidence intervals (CIs) for each outcome.

To evaluate heterogeneity, we used Cochran’s Q test, Higgins’ I^2^, τ using the DerSimonian–Laird estimator, and the prediction interval (PI) methods to assess heterogeneity. The PI was not calculated if τ = 0.0. Heterogeneity was considered substantial if Cochran’s Q test yielded a p-value < 0.1 or if the I² value exceeded 50%. We used a random-effects meta-analysis to combine the data, as we expected clinical heterogeneity to be sufficient to predict that the underlying treatment effects differed between trials.

For heterogeneous outcomes, sensitivity analysis was conducted by removing one study at a time to identify its impact on the overall results. The mean and standard deviation (SD) were calculated when outcomes were reported as median (P_25_–P_75_), median (range), or mean (standard error of the mean). For outcomes measured at multiple time points, we combined data across all time points for analysis, as these measurements were interdependent, and conducting multiple comparisons would increase the risk of Type I error.

**Trial sequential analysis**

Random errors due to limited data are associated with conventional meta-analyses. To address this, TSA was performed to determine the required information size (RIS) and establish a threshold for statistical significance by controlling the risk of potential false-positive and -negative results. A random-effects model was used to construct a cumulative Z-curve. The TSA was conducted to maintain an overall 5% risk of Type I errors.

To control the overall Type I error rate at 5%, O'Brien–Fleming-type monitoring boundaries were constructed to define thresholds for statistical significance (benefit or harm) and futility. When the cumulative Z-curve crossed the trial sequential monitoring boundary or entered the futility area, it indicated that the evidence was sufficient to accept or reject the anticipated intervention effect, with no further studies required. If the Z-curve did not cross any boundaries and the Required Information Size (RIS) was not reached, the evidence was considered inconclusive, indicating the need for further studies.

For dichotomous outcomes, the RIS was estimated based on the observed proportion of patients who had an outcome in the propofol group (the cumulative proportion of patients who had an event relative to the number of patients in the propofol group), an RR reduction of 30% in the remimazolam group, an alpha of 5% for all outcomes, a beta of 20%, and observed diversity based on the included trials. For quantitative outcomes, we used the observed SD in the trial sequential analysis, the mean difference of the observed SD/3, an alpha of 5% for all outcomes, a beta of 10%, and the observed diversity based on the included trials.

**Supplementary document 3.** Methodological assessment of risk of bias and evidence quality using RoB 2.0 and GRADE

**Risk of Bias assessment**

The risk of bias for each included study was independently assessed by two investigators using the Revised Cochrane Risk of Bias Tool for Randomized Trials (RoB 2.0). This tool evaluates bias across five domains: (1) bias arising from the randomization process; (2) bias due to deviations from the intended interventions; (3) bias due to missing outcome data; (4) bias in the measurement of the outcome; and (5) bias in selection of the reported result. An overall risk of bias was assigned based on the domain-level assessments: 1) Low risk of bias: All domains were rated as low risk, 2) High risk of bias: At least one domain was rated as high risk or multiple domains were rated as having some concerns, and 3) Some concerns: The overall risk did not meet the criteria for low or high risk.

**Quality of the Evidence assessment**

The guidelines of the Grading of Recommendations, Assessment, Development, and Evaluation (GRADE) system were applied to evaluate the quality of evidence. The guidelines involve the sequential assessment of the evidence quality, evaluation of the risk–benefit balance, and subsequent appraisal of the strength of the recommendations. The evidence quality was graded as follows: (1) high, indicating that the confidence in the effect estimate was unlikely to alter with further research; (2) moderate, indicating that further research was likely to significantly alter confidence in the effect estimate and might alter the estimate; (3) low, indicating that further research was likely to significantly change confidence in the effect estimate and alter the estimate; and (4) very low, indicating that no effect estimate was certain.

**Supplementary Figure S1. Forest plot for hypertension comparing remimazolam and propofol.** The figure depicts individual trials as filled squares with relative sample size and the 95% confidence interval (CI) of the difference as a solid line. The diamond shape indicates the pooled estimate and uncertainty for the combined effect. The pooled estimate indicates no significant difference in the incidence of hypertension between remimazolam and propofol.


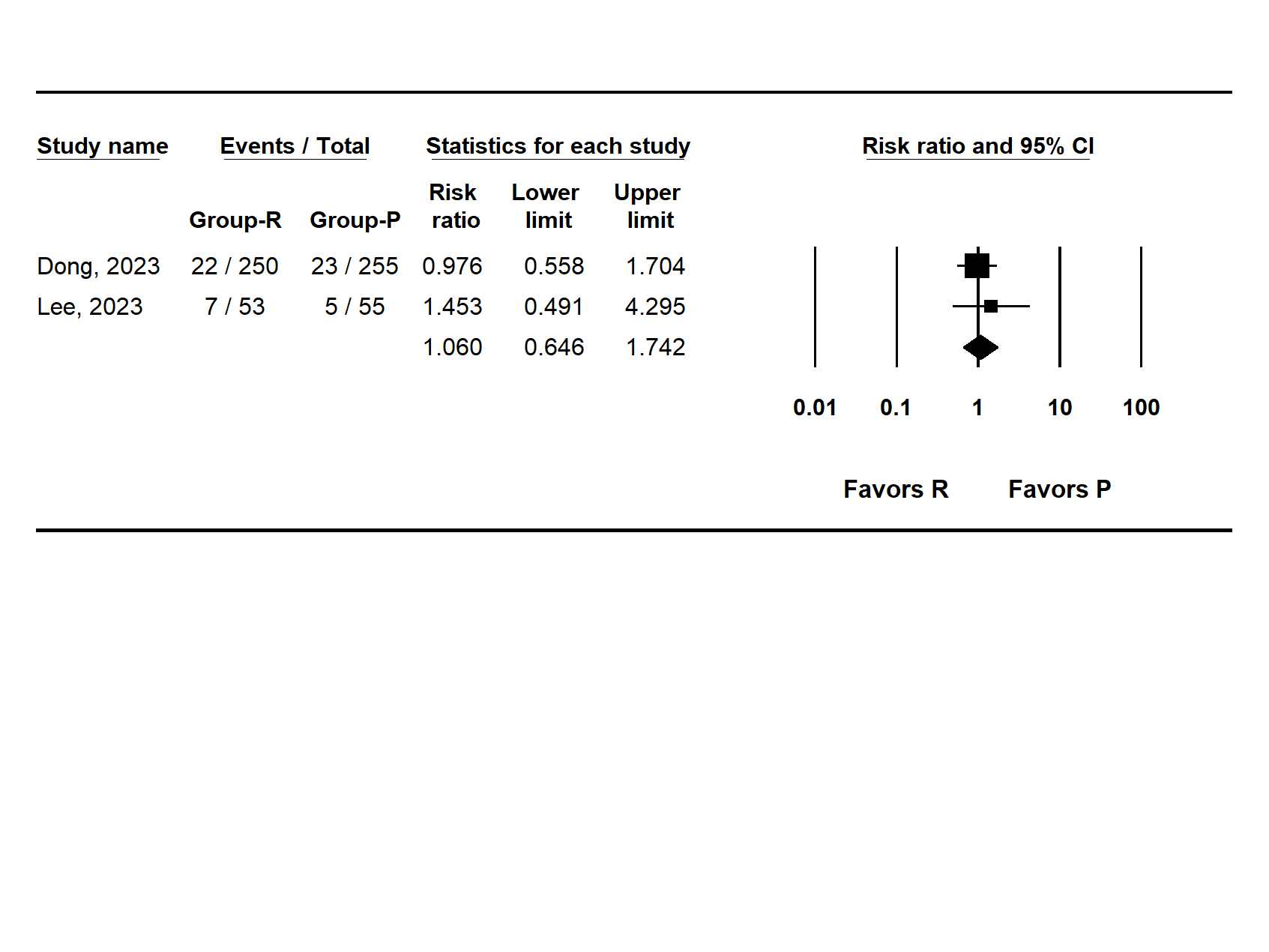


**Supplementary Figure S2. Trial sequential analysis plot for hypertension comparing remimazolam and propofol.** Uppermost and lowermost complete red curves represent trial sequential monitoring boundary lines for benefit and harm respectively. Horizontal dotted red line represents the conventional boundaries for statistical significance. Triangular red lines on the right side reflects the futility boundaries. The blue solid line represents the cumulative z-curve. The number on the x-axis indicates required information size (n=3043). The TSA suggests insufficient evidence, with only 20.1 % of the required information size (RIS) accrued, as the Z-curve crossed neither the conventional test boundary nor cross the trial sequential monitoring boundary.


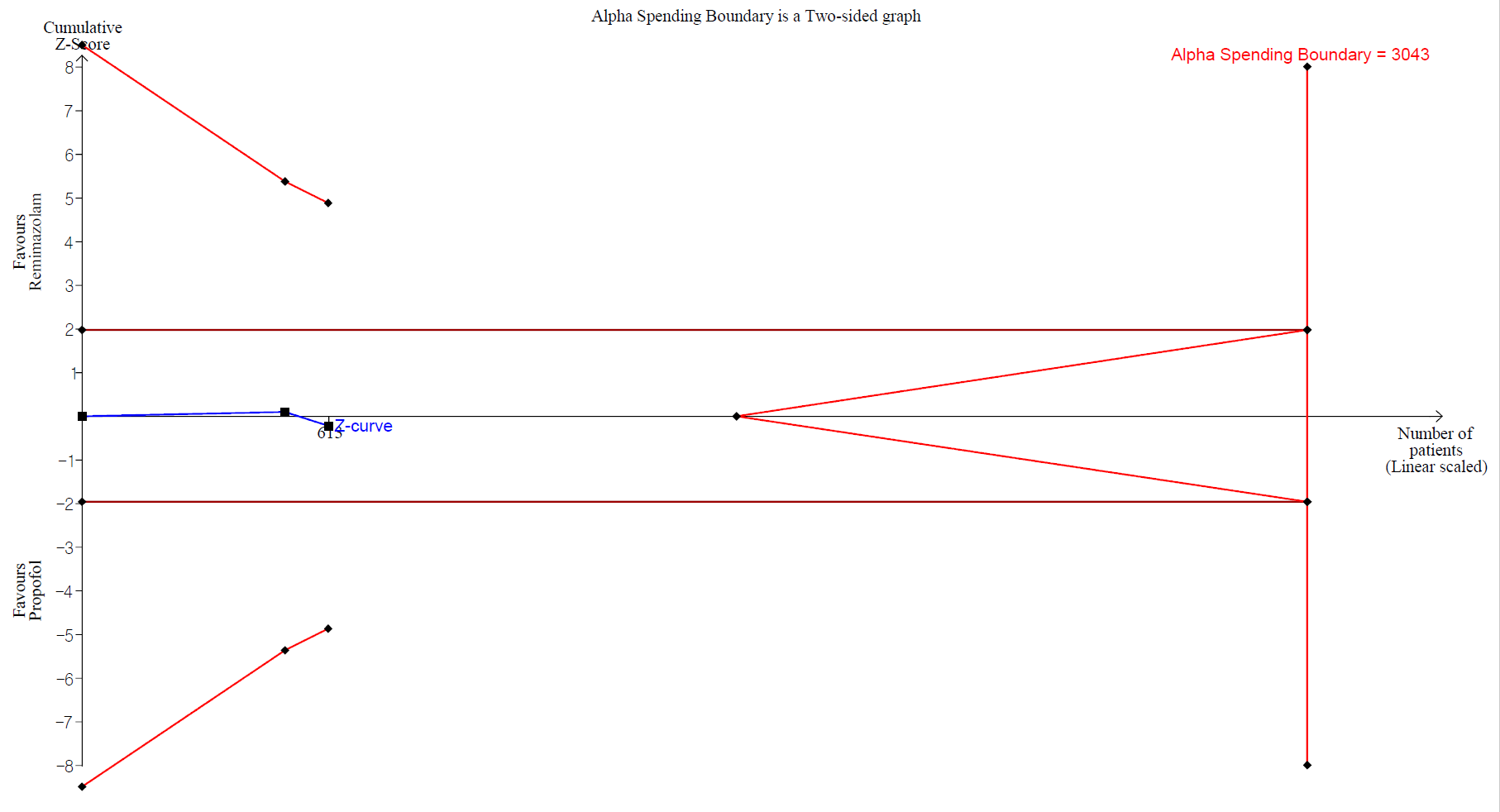


**Supplementary Figure S3. Forest plot for tachycardia comparing remimazolam and propofol.** The figure depicts individual trials as filled squares with relative sample size and the 95% confidence interval (CI) of the difference as a solid line. The diamond shape indicates the pooled estimate and uncertainty for the combined effect. The pooled estimate indicates significant difference in the incidence of tachycardia between remimazolam and propofol.


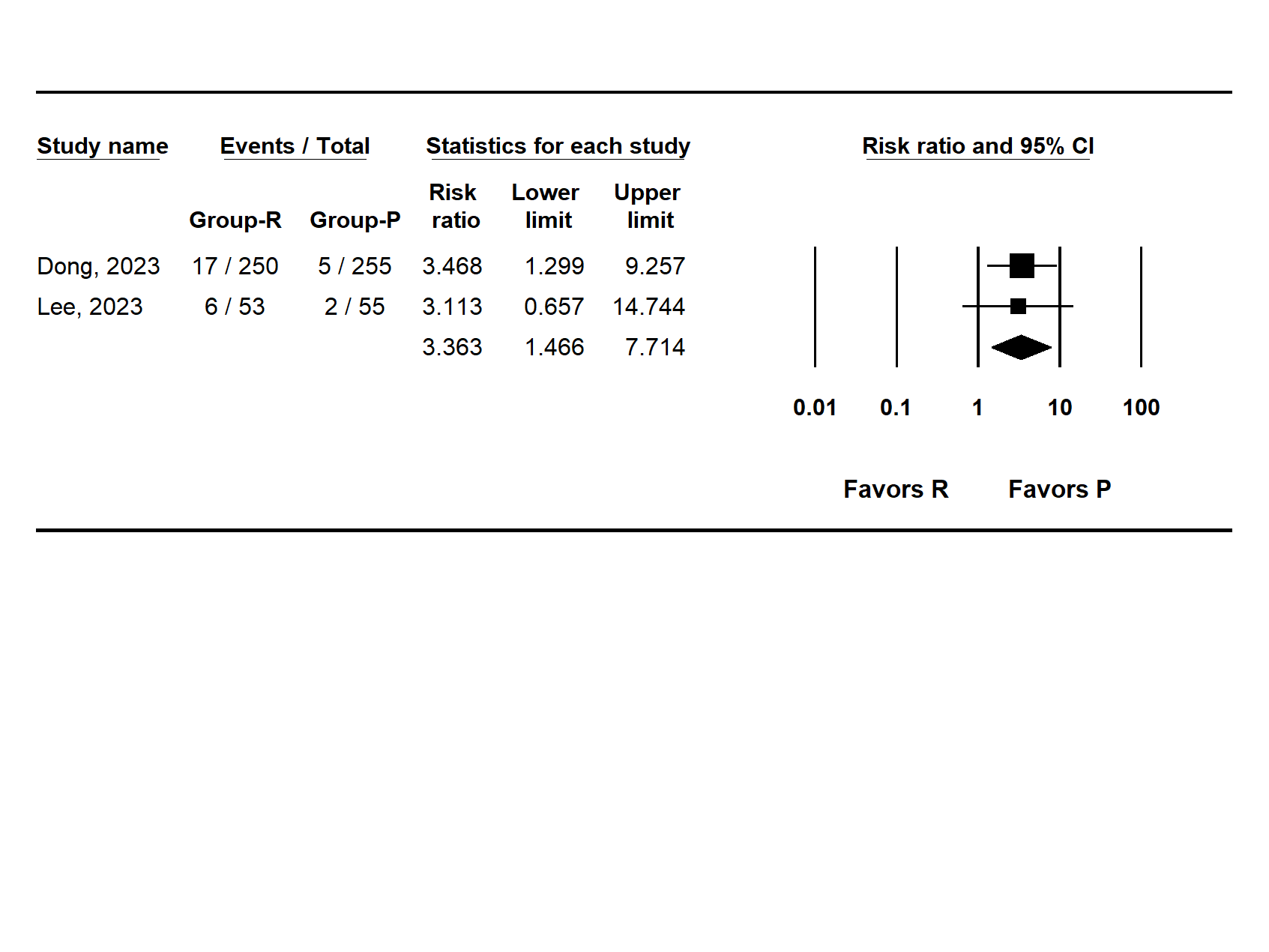


**Supplementary Figure S4. Trial sequential analysis plot for tachycardia comparing remimazolam and propofol.** Horizontal dotted red line represents the conventional boundaries for statistical significance. The blue solid line represents the cumulative z-curve. The TSA suggests insufficient evidence, with only 4.8% (613 of 12640 patients) of the required information size (RIS) accrued, as the Z curve crossed the conventional test boundary, but did not cross the trial sequential monitoring boundary.


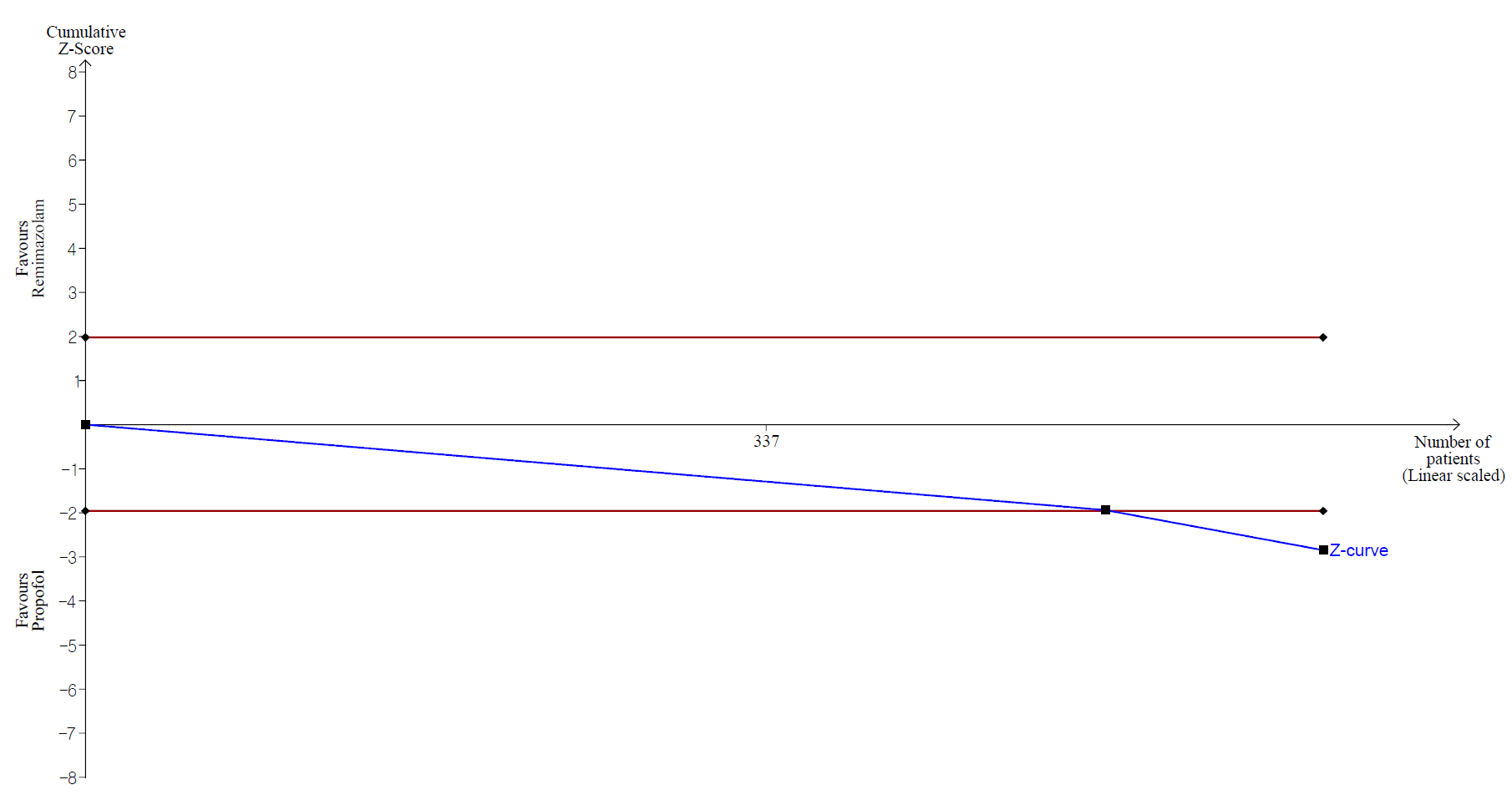


**Supplementary Figure S5. Forest plot for MAP comparing remimazolam and propofol.** The figure depicts individual trials as filled squares with relative sample size and the 95% confidence interval (CI) of the difference as a solid line. The diamond shape indicates the pooled estimate and uncertainty for the combined effect. The pooled estimate indicates no significant difference in intraoperative MAP between remimazolam and propofol.


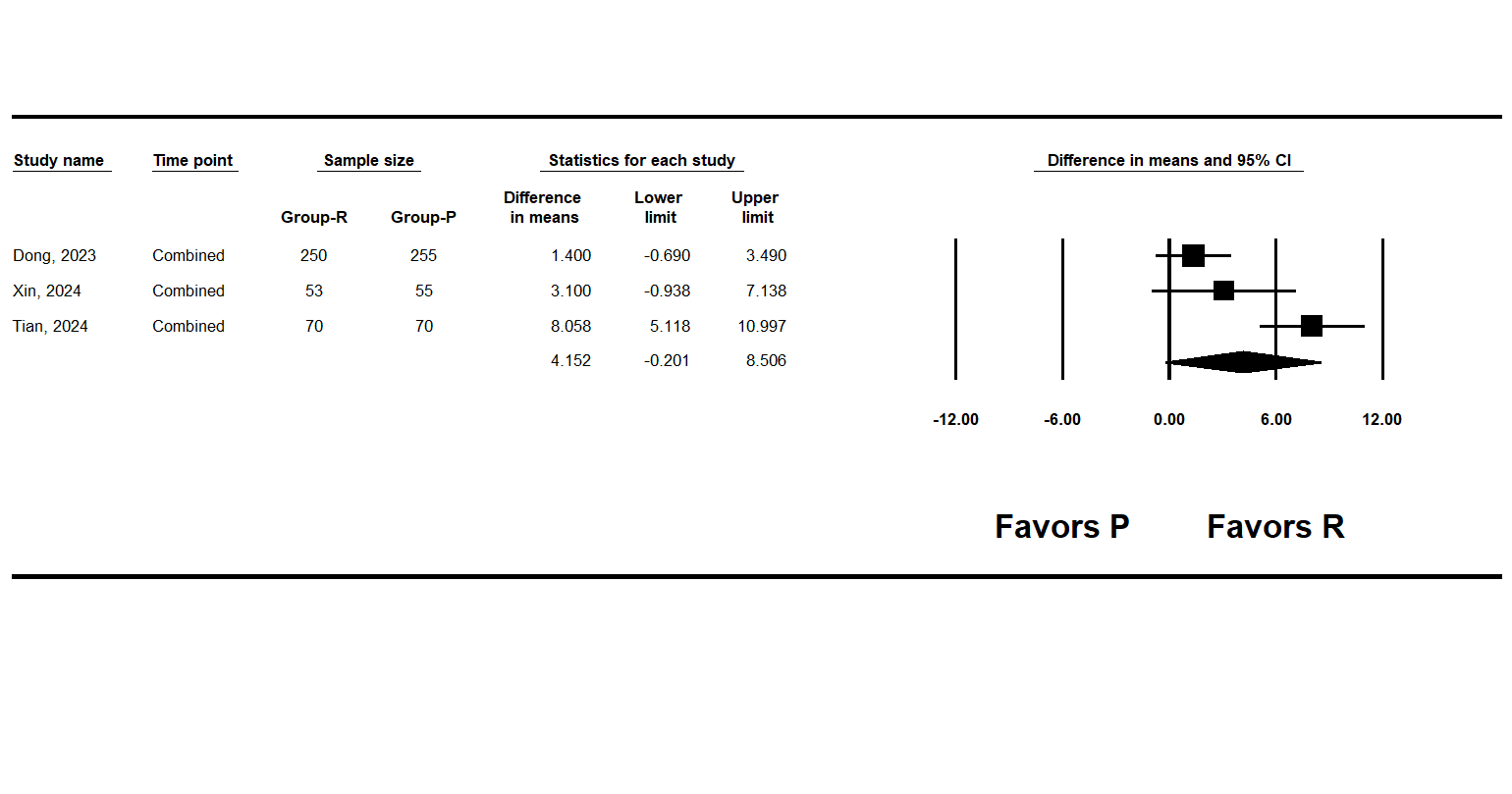


**Supplementary Figure S6. Forest plot for HR comparing remimazolam and propofol.** The figure depicts individual trials as filled squares with relative sample size and the 95% confidence interval (CI) of the difference as a solid line. The diamond shape indicates the pooled estimate and uncertainty for the combined effect. The pooled estimate indicates significant difference in intraoperative HR between remimazolam and propofol.


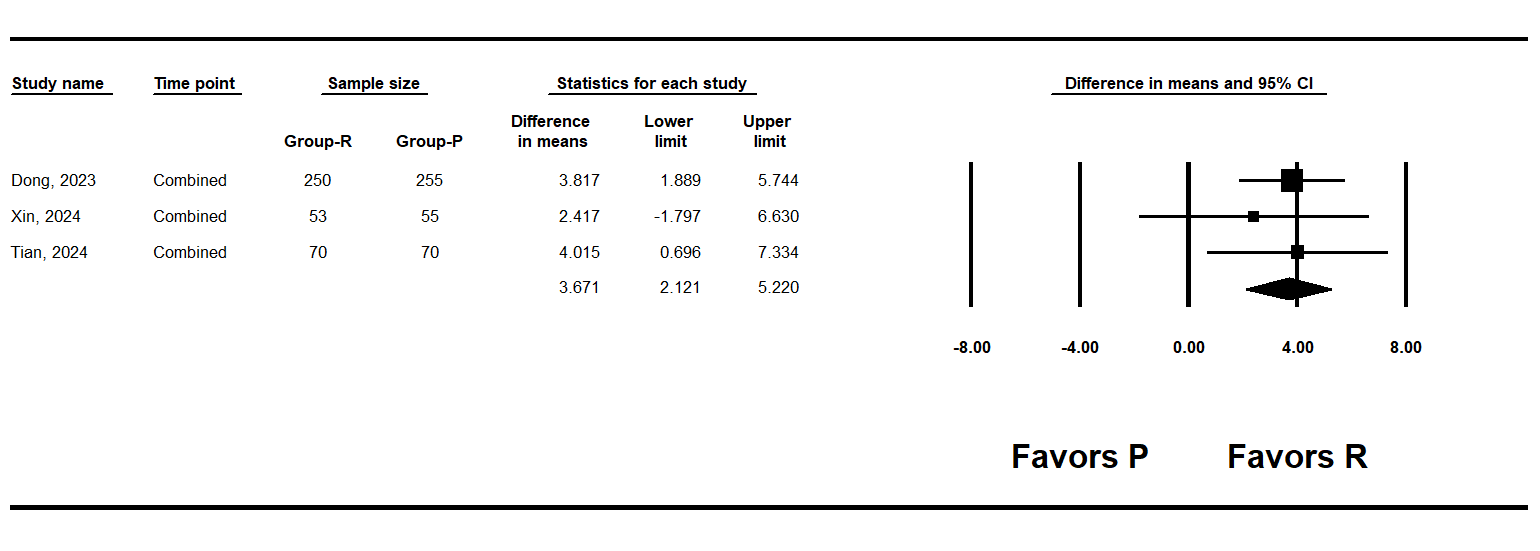


**Supplementary Figure S7. Forest plot for completion rate comparing remimazolam and propofol.** The figure depicts individual trials as filled squares with relative sample size and the 95% confidence interval (CI) of the difference as a solid line. The diamond shape indicates the pooled estimate and uncertainty for the combined effect. The pooled estimate indicates no significant difference in completion rate between remimazolam and propofol.


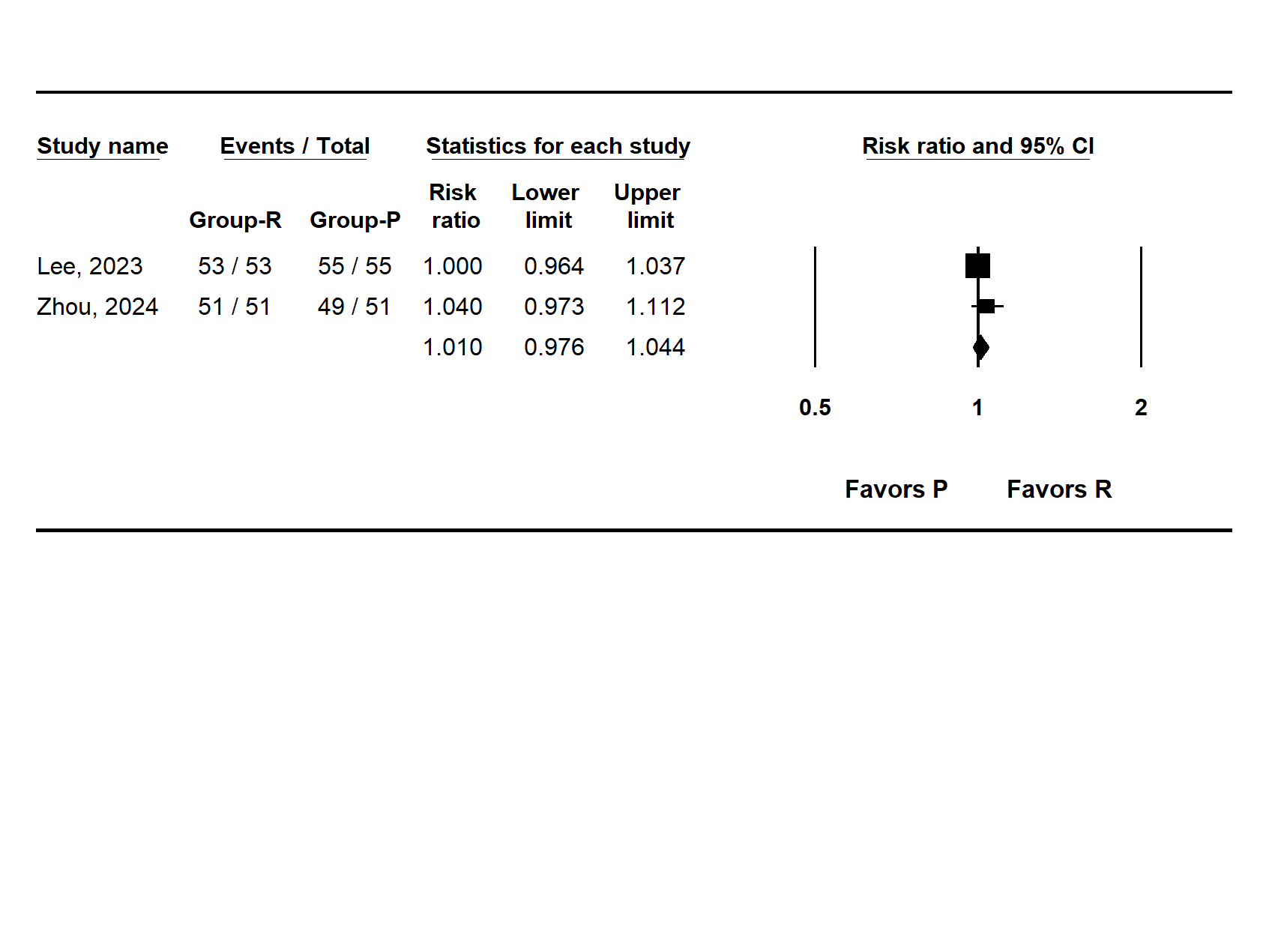


**Supplementary Figure S8. Trial sequential analysis plot for completion rate comparing remimazolam and propofol.** Uppermost and lowermost complete red curves represent trial sequential monitoring boundary lines for benefit and harm respectively. Horizontal dotted red line represents the conventional boundaries for statistical significance. Triangular red lines on the right side reflects the futility boundaries. The blue solid line represents the cumulative z-curve. The number on the x-axis indicates required information size (n=659). The TSA suggests insufficient evidence, with only 31.9 % of the required information size (RIS) accrued, as the Z-curve crossed neither the conventional test boundary nor cross the trial sequential monitoring boundary.


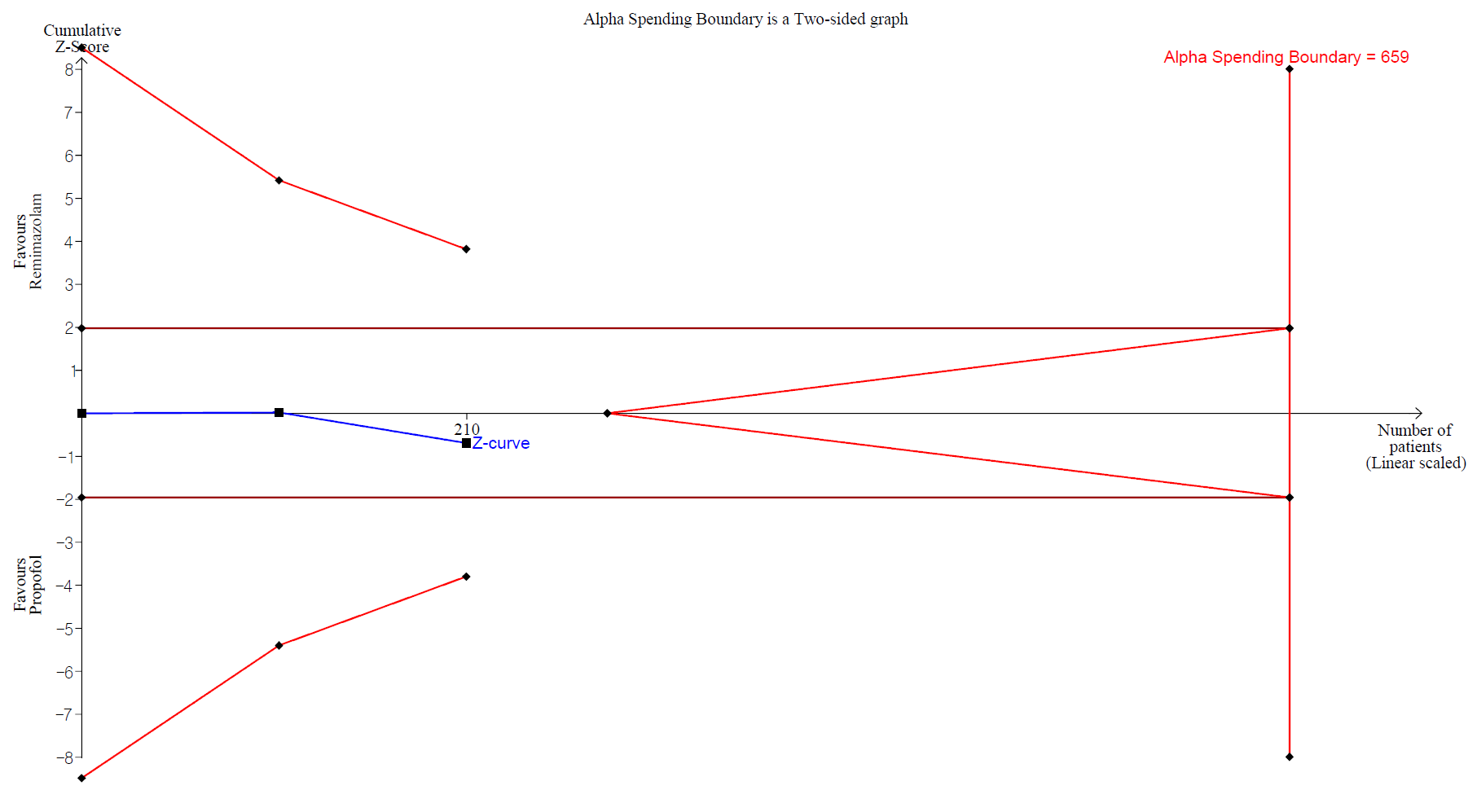


**Supplementary Figure S9. Forest plot for body movement comparing remimazolam and propofol.** The figure depicts individual trials as filled squares with relative sample size and the 95% confidence interval (CI) of the difference as a solid line. The diamond shape indicates the pooled estimate and uncertainty for the combined effect. The pooled estimate indicates significant difference in body movement between remimazolam and propofol.


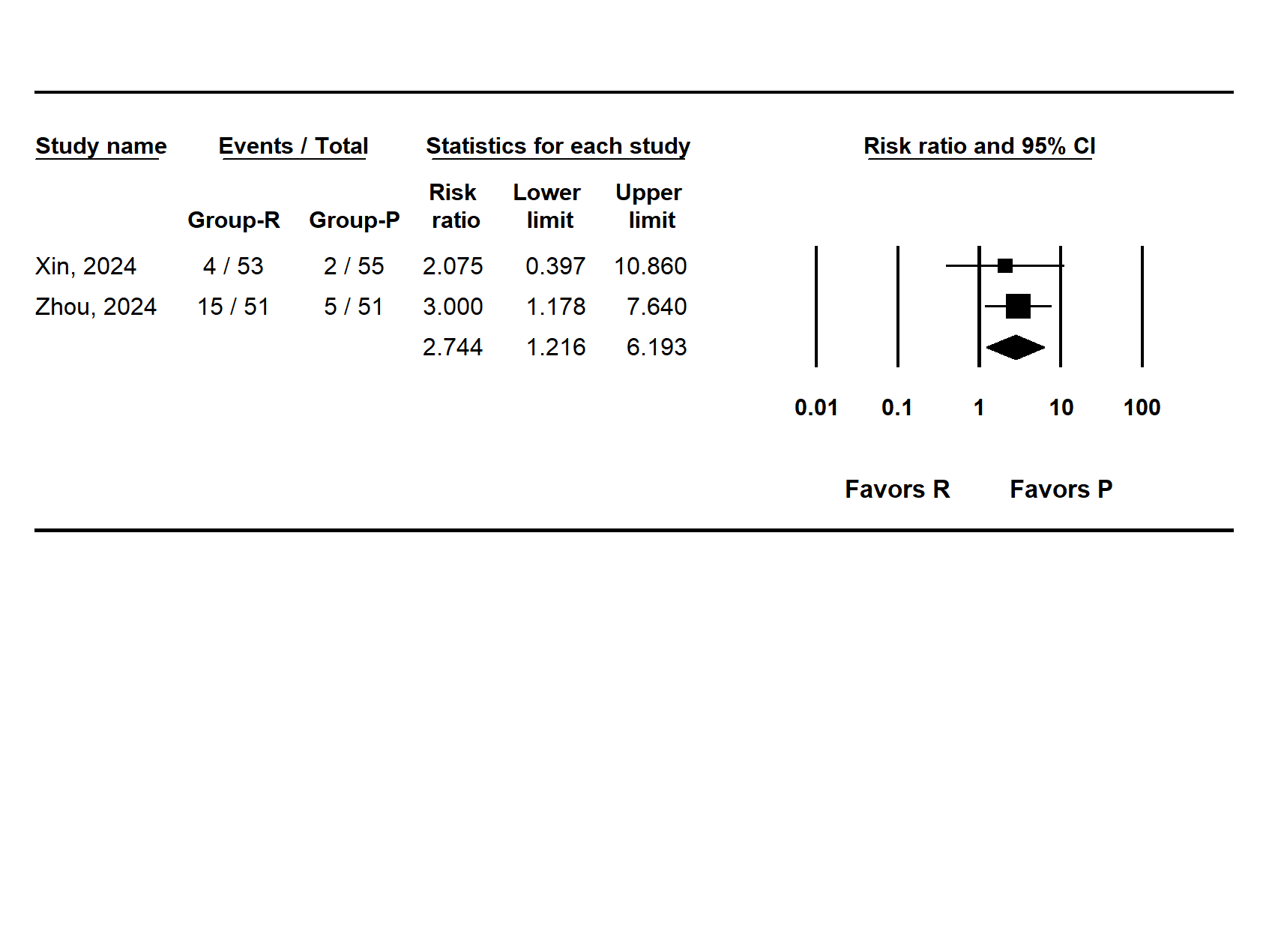


**Supplementary Figure S10. Trial sequential analysis plot for body movement comparing remimazolam and propofol.** Horizontal dotted red line represents the conventional boundaries for statistical significance. Triangular red lines on the right side reflects the futility boundaries. The blue solid line represents the cumulative z-curve. The number on the x-axis indicates required information size (n=4174). The TSA suggests insufficient evidence, with only 5.0 % of the required information size (RIS) accrued, as the Z-curve crossed the conventional test boundary but did not cross the trial sequential monitoring boundary.


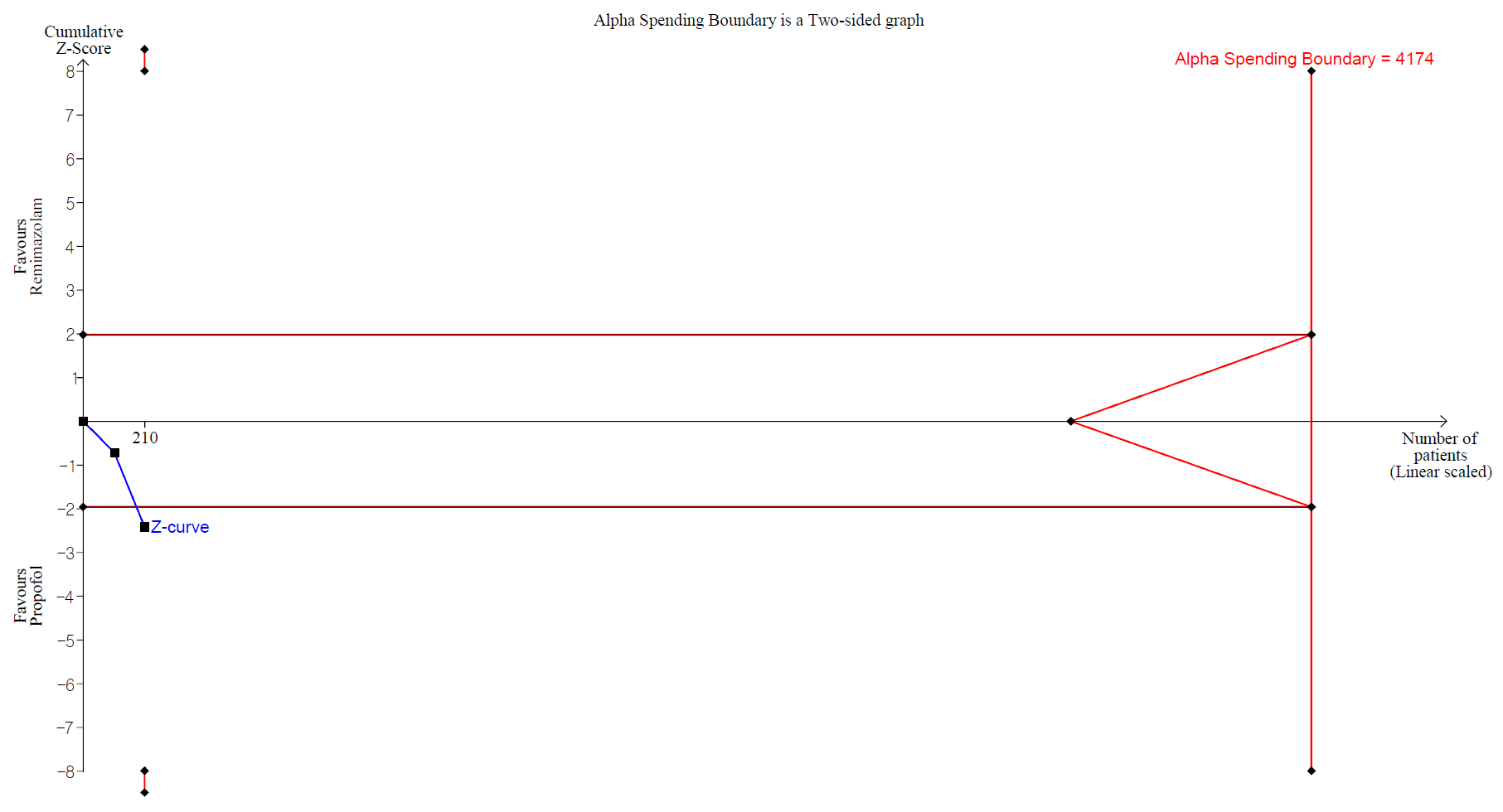


**Supplementary Figure S11. Forest plot for BIS comparing remimazolam and propofol.** The figure depicts individual trials as filled squares with relative sample size and the 95% confidence interval (CI) of the difference as a solid line. The diamond shape indicates the pooled estimate and uncertainty for the combined effect. The pooled estimate indicates significant difference in BIS between remimazolam and propofol.


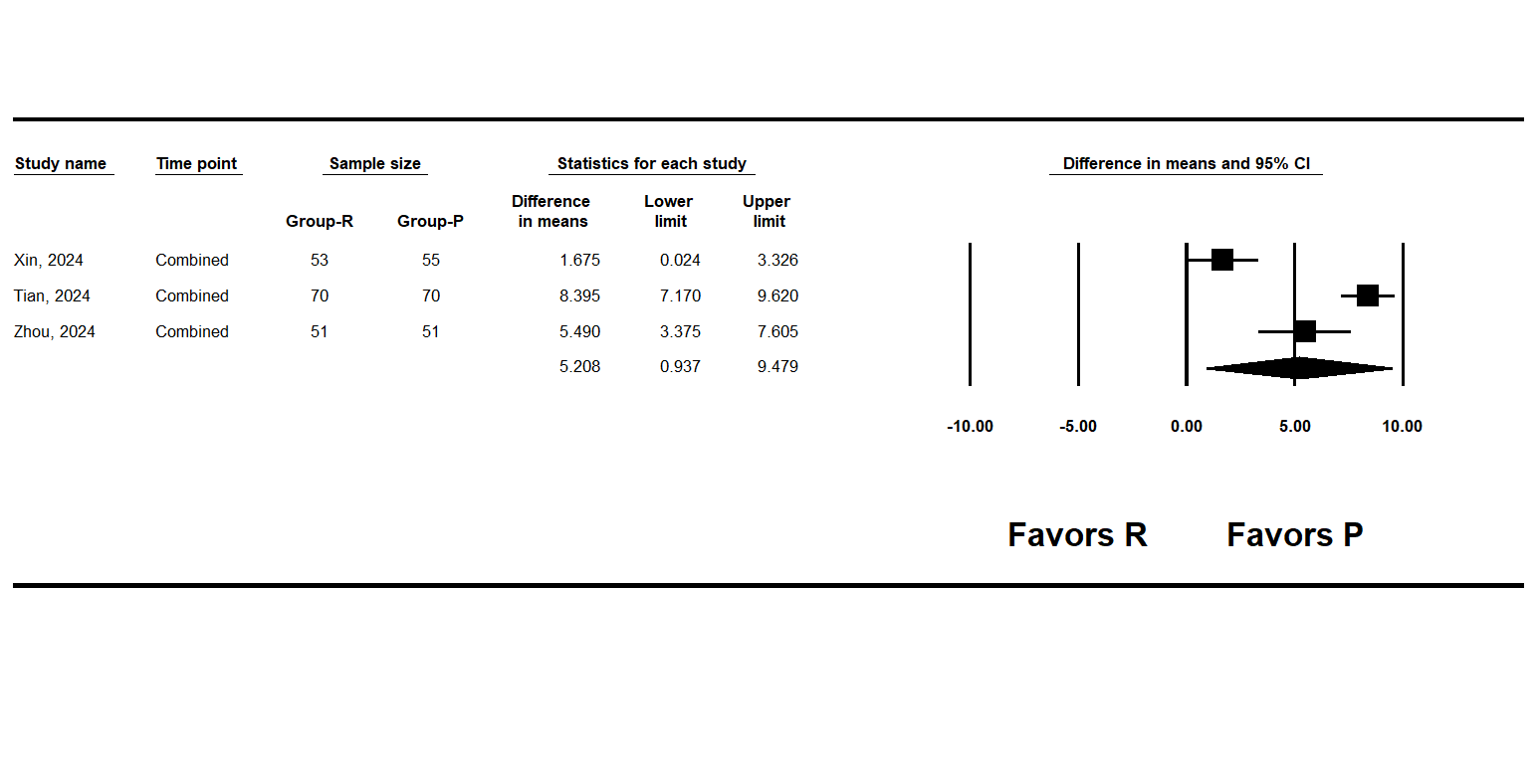


**Supplementary Figure S12. Forest plot for agitation and delirium comparing remimazolam and propofol.** The figure depicts individual trials as filled squares with relative sample size and the 95% confidence interval (CI) of the difference as a solid line. The diamond shape indicates the pooled estimate and uncertainty for the combined effect. The pooled estimate indicates significant difference in injection pain between remimazolam and propofol.

**
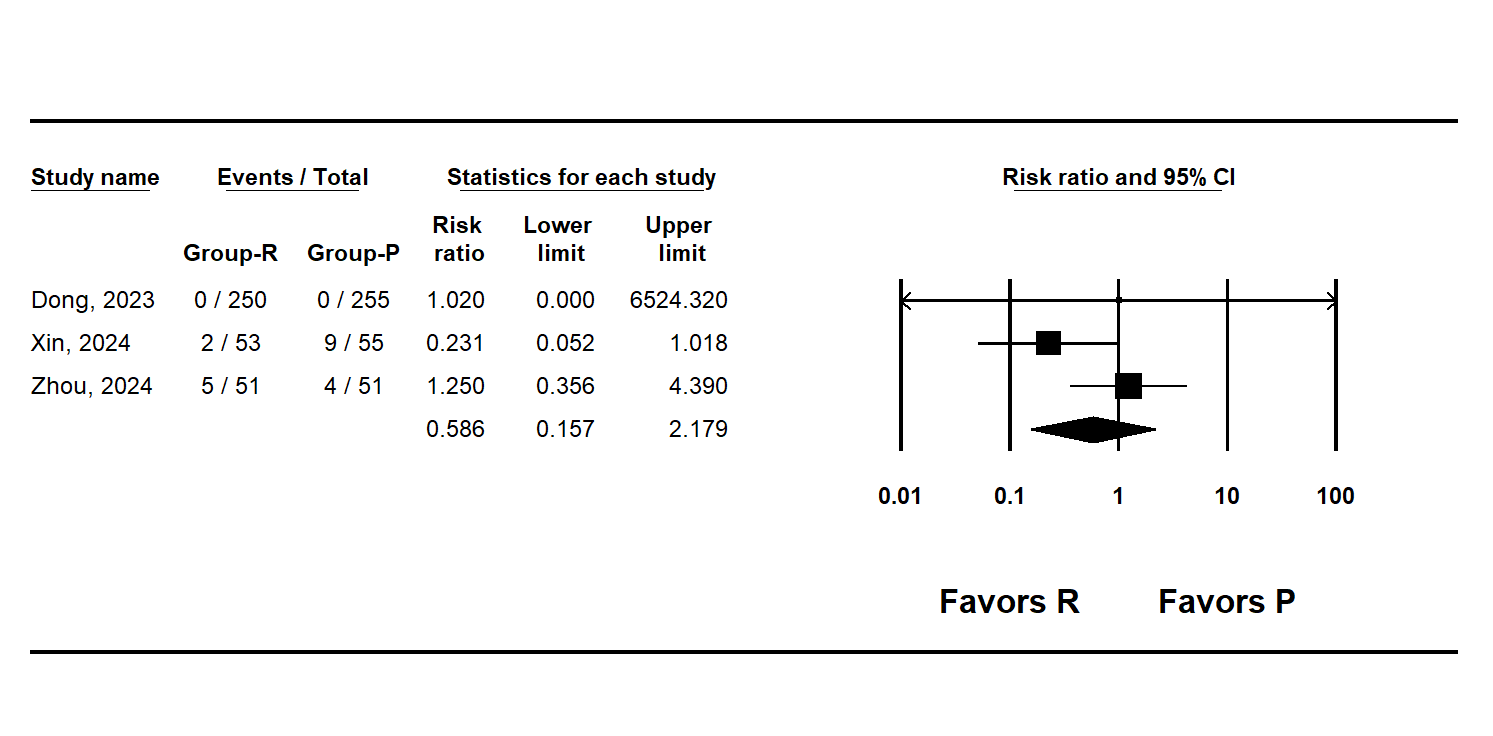
**

**Supplementary Figure S13. Trial sequential analysis plot for agitation and delirium comparing remimazolam and propofol.** Horizontal dotted red line represents the conventional boundaries for statistical significance. The blue solid line represents the cumulative z-curve. The TSA suggests insufficient evidence, with only 2.9% (715 of 24612 patients) of the required information size (RIS) accrued, as the Z-curve crossed neither the conventional test boundary nor cross the trial sequential monitoring boundary.


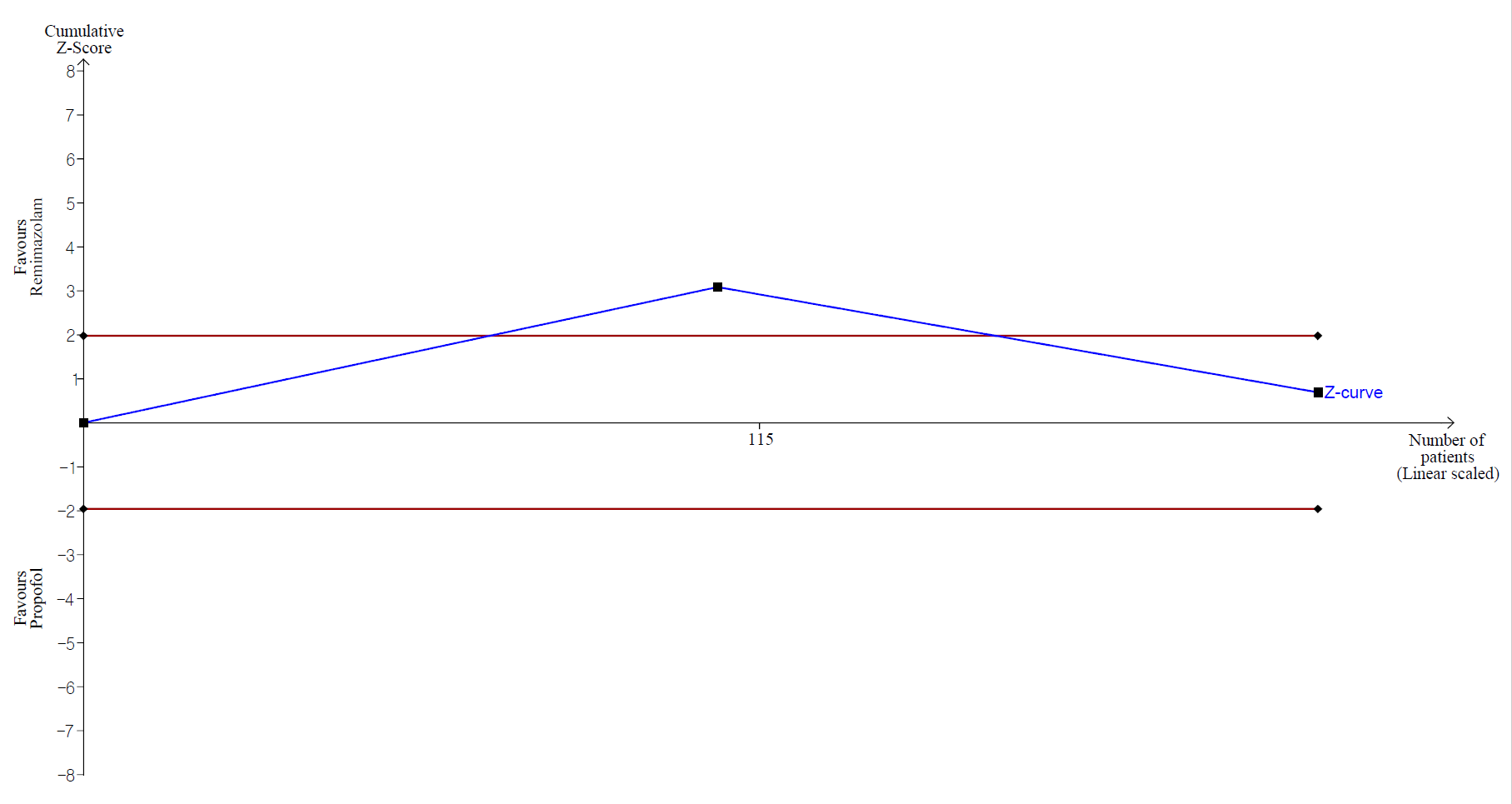


**Supplementary Figure S14. Forest plot for POD1 QOR15 comparing remimazolam and propofol.** The figure depicts individual trials as filled squares with relative sample size and the 95% confidence interval (CI) of the difference as a solid line. The diamond shape indicates the pooled estimate and uncertainty for the combined effect. The pooled estimate indicates no significant difference in PONV between remimazolam and propofol.


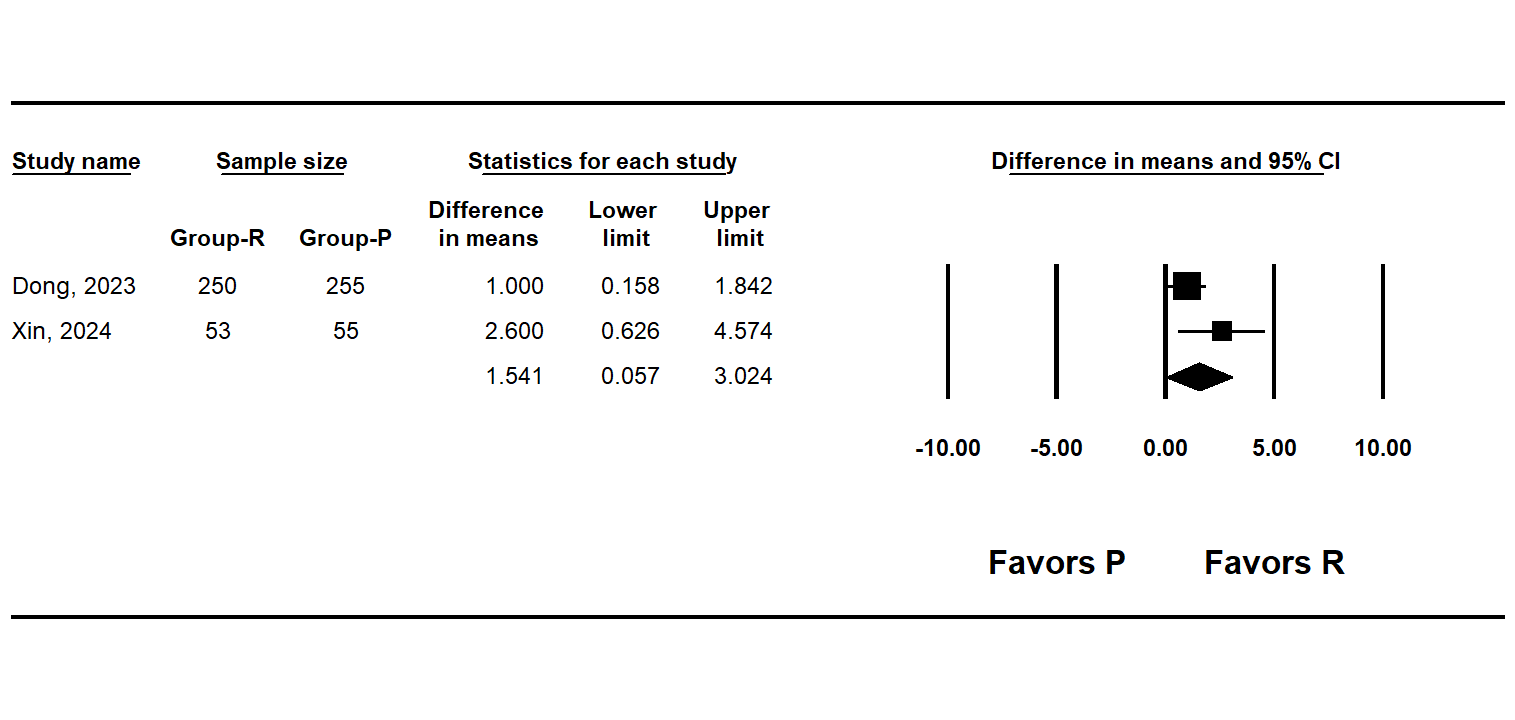


**Supplementary Figure S15. Trial sequential analysis plot for POD1 QOR15 comparing remimazolam and propofol.** Uppermost and lowermost complete red curves represent trial sequential monitoring boundary lines for benefit and harm respectively. Horizontal dotted red line represents the conventional boundaries for statistical significance. Triangular red lines on the right side reflects the futility boundaries. The blue solid line represents the cumulative z-curve. The number on the x-axis indicates required information size (n=1038). The TSA suggests insufficient evidence, with only 59.1 % of the required information size (RIS) accrued, as the Z-curve crossed the conventional test boundary but did not cross the trial sequential monitoring boundary.


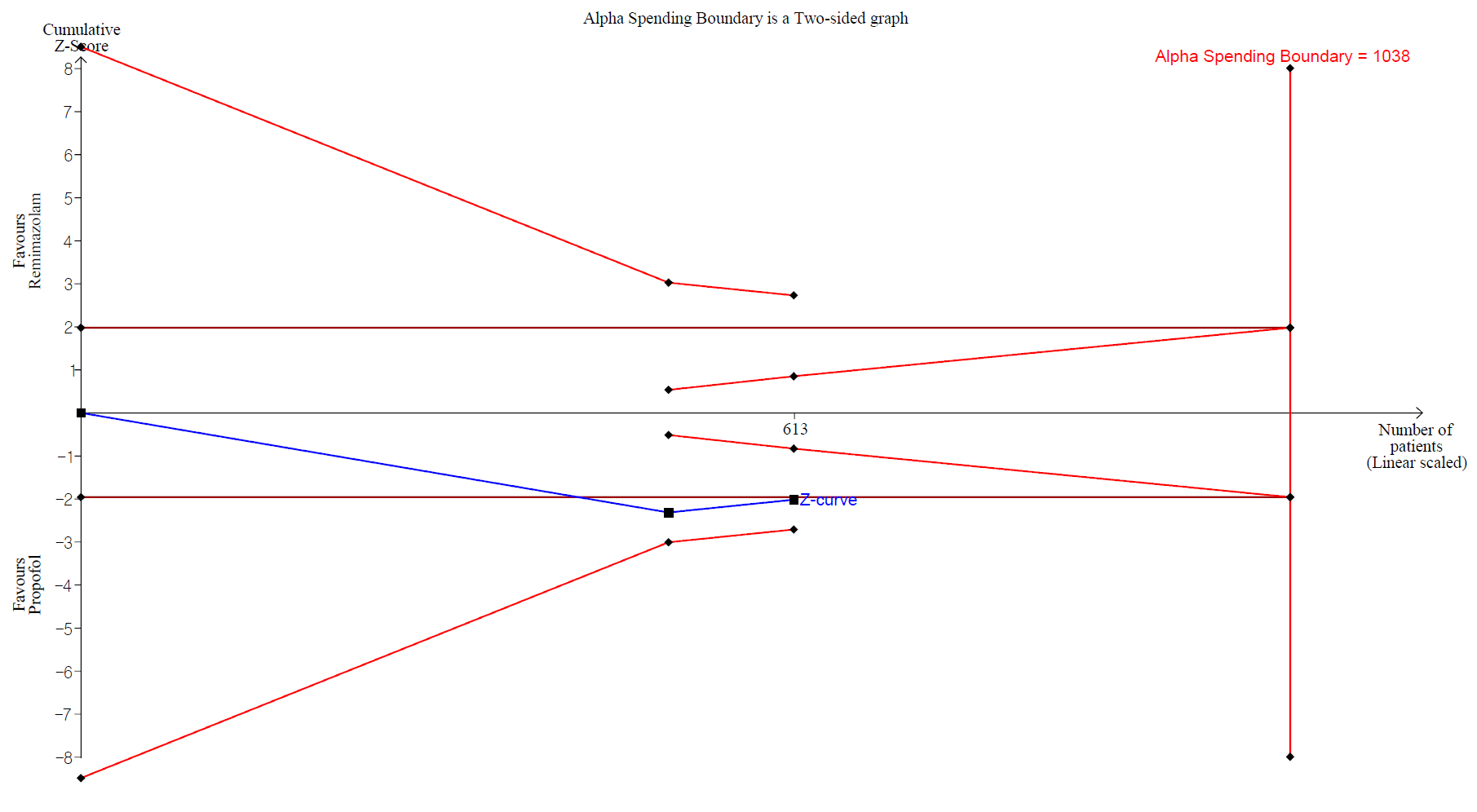


**Supplementary Figure S16. Forest plot for procedure time comparing remimazolam and propofol.** The figure depicts individual trials as filled squares with relative sample size and the 95% confidence interval (CI) of the difference as a solid line. The diamond shape indicates the pooled estimate and uncertainty for the combined effect. The pooled estimate indicates no significant difference in procedure time between remimazolam and propofol.


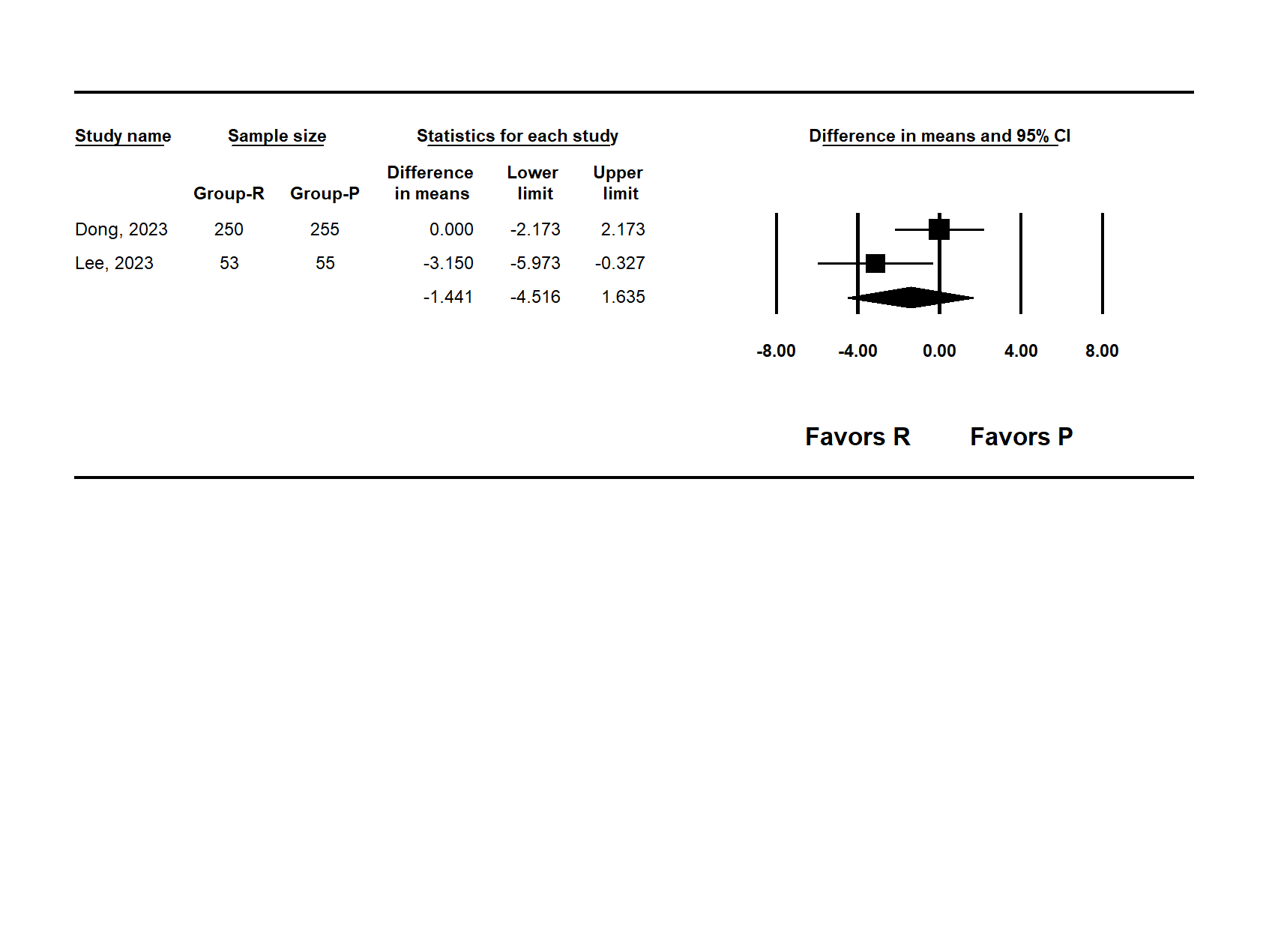


**Supplementary Figure S17. Trial sequential analysis plot for procedure time comparing remimazolam and propofol.** Uppermost and lowermost complete red curves represent trial sequential monitoring boundary lines for benefit and harm respectively. Horizontal dotted red line represents the conventional boundaries for statistical significance. Triangular red lines on the right side reflects the futility boundaries. The blue solid line represents the cumulative z-curve. The number on the x-axis indicates required information size (n=902). The TSA suggests insufficient evidence, with only 68.0 % of the required information size (RIS) accrued. But, the cumulative Z curve crossed the futility boundary, suggesting no further studies were needed.


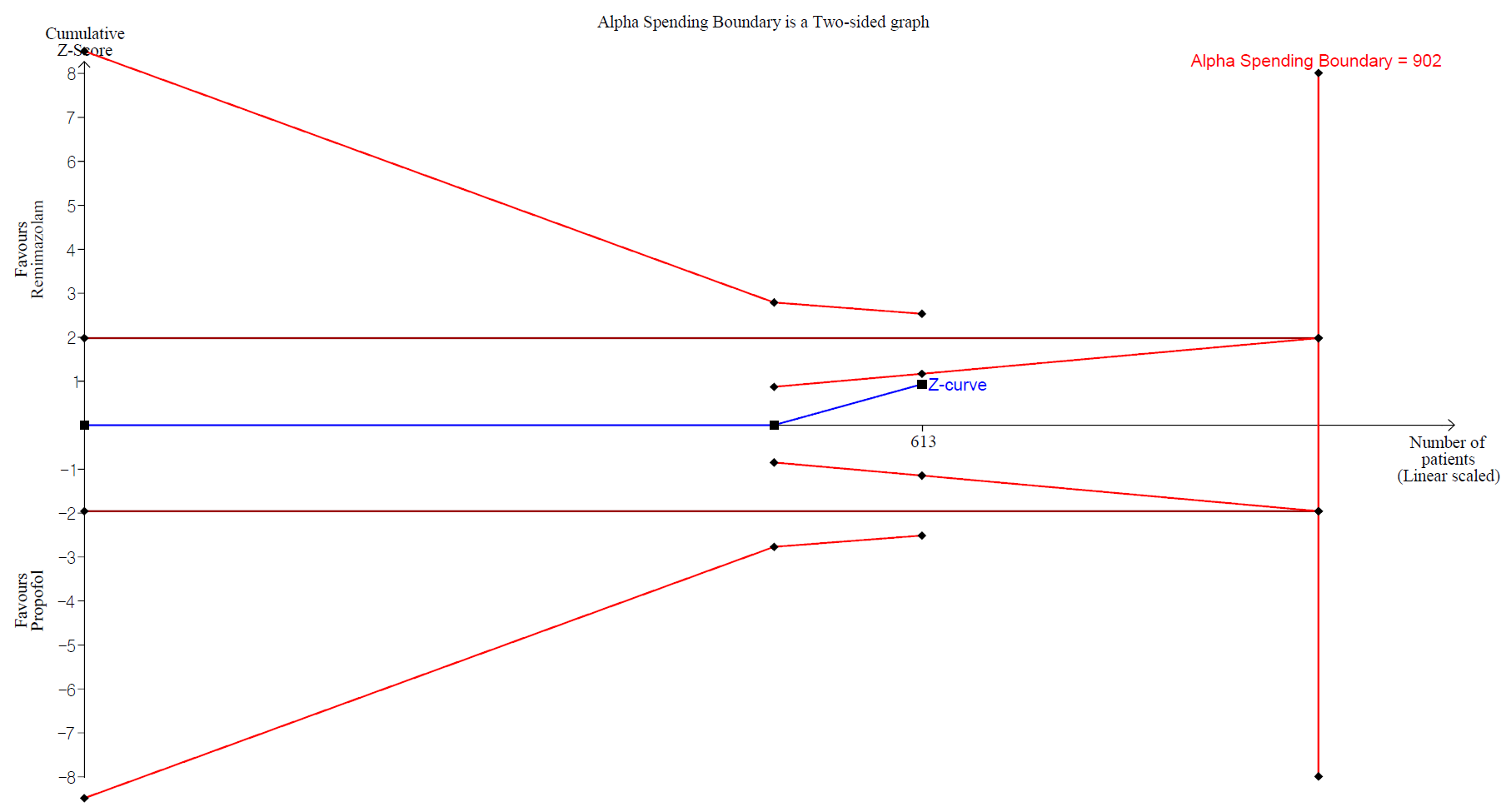


**Supplementary Figure S18. Forest plot for induction time comparing remimazolam and propofol.** The figure depicts individual trials as filled squares with relative sample size and the 95% confidence interval (CI) of the difference as a solid line. The diamond shape indicates the pooled estimate and uncertainty for the combined effect. The pooled estimate indicates no significant difference in induction time between remimazolam and propofol.


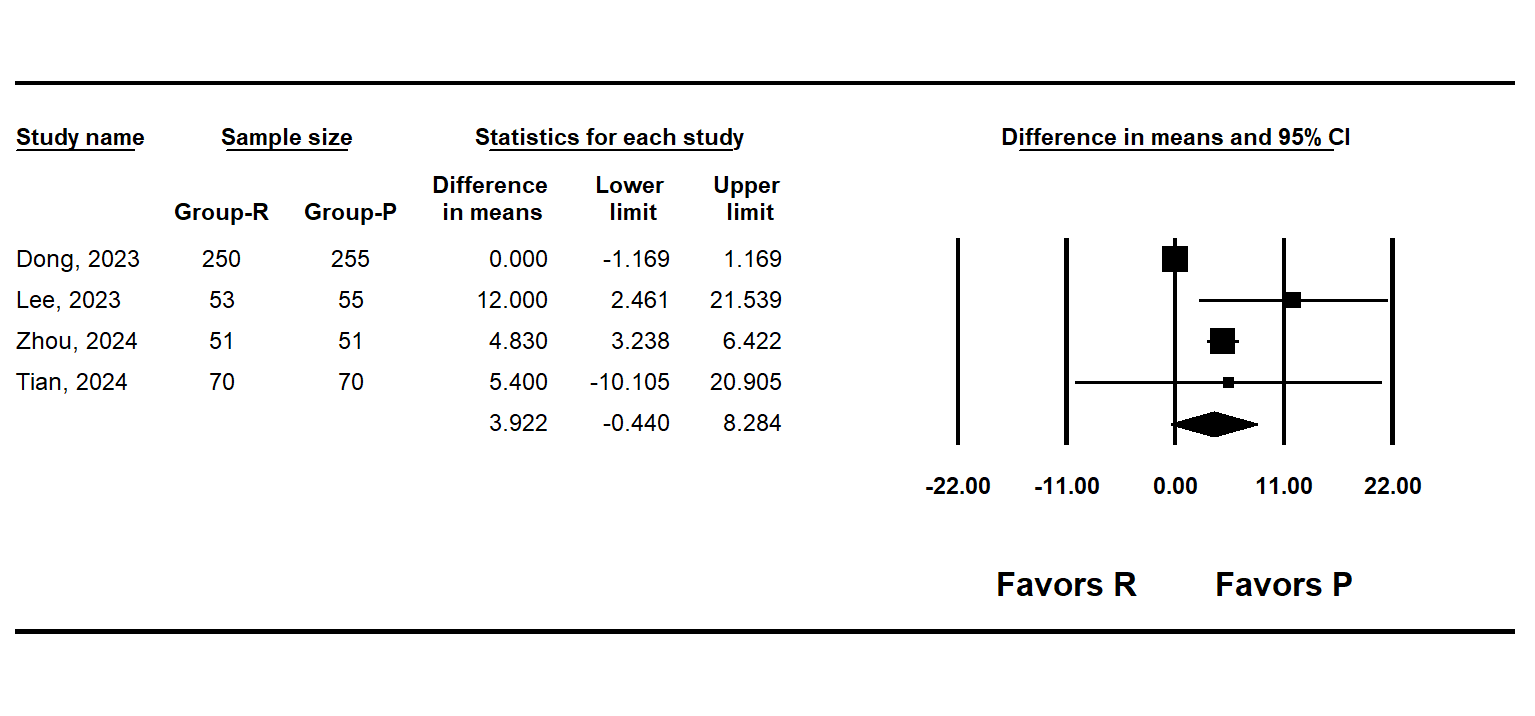


**Supplementary Figure S19. Trial sequential analysis plot for induction time comparing remimazolam and propofol.** Uppermost and lowermost complete red curves represent trial sequential monitoring boundary lines for benefit and harm respectively. Horizontal dotted red line represents the conventional boundaries for statistical significance. Triangular red lines on the right side reflects the futility boundaries. The blue solid line represents the cumulative z-curve. The number on the x-axis indicates required information size (n=6120). The TSA suggests insufficient evidence, with only 14.0 % of the required information size (RIS) accrued, as the Z-curve crossed neither the conventional test boundary nor cross the trial sequential monitoring boundary.


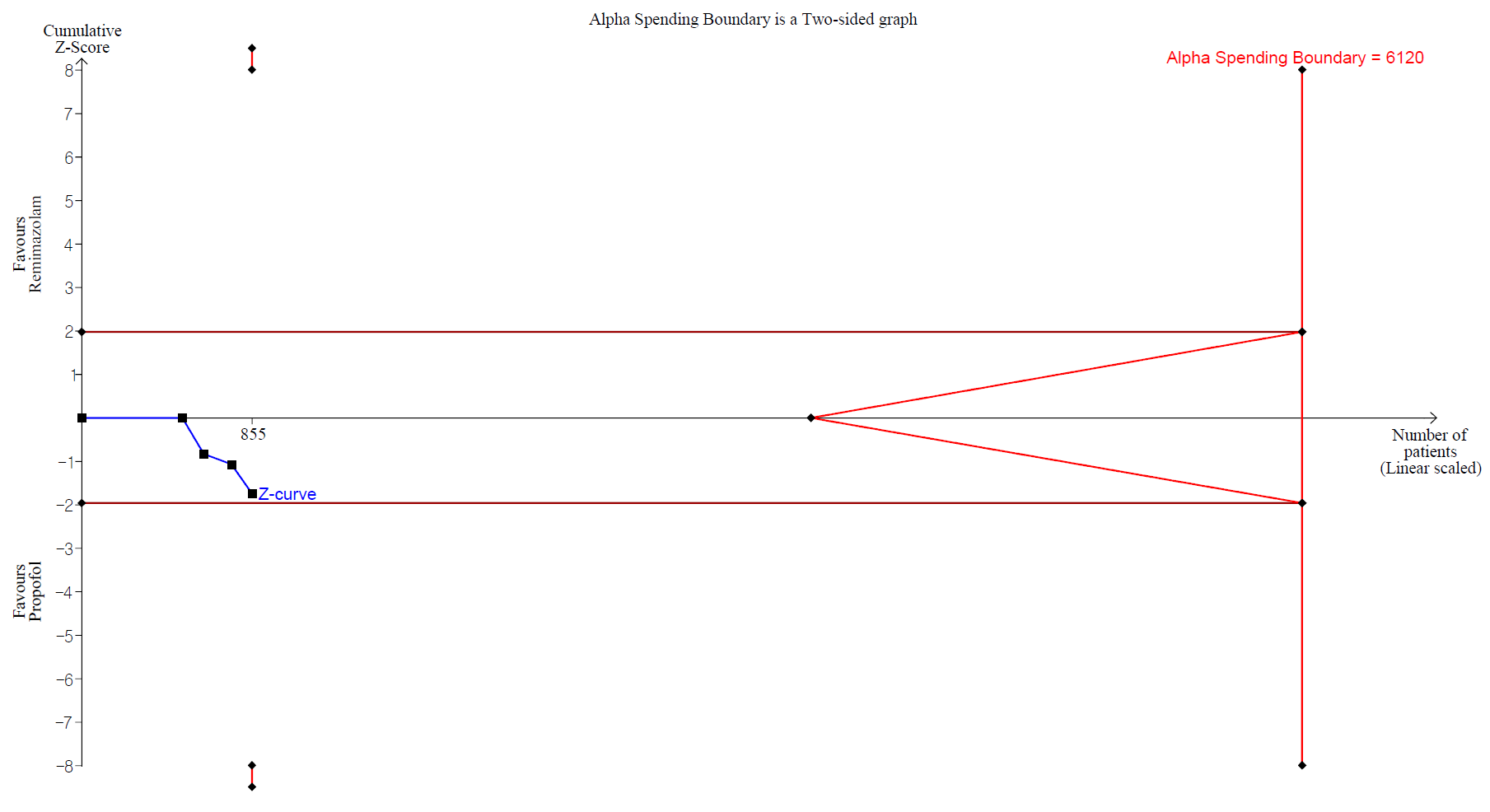


**Supplementary Figure S20. Forest plot for awake time comparing remimazolam and propofol.** The figure depicts individual trials as filled squares with relative sample size and the 95% confidence interval (CI) of the difference as a solid line. The diamond shape indicates the pooled estimate and uncertainty for the combined effect. The pooled estimate indicates no significant difference in awake time between remimazolam and propofol.


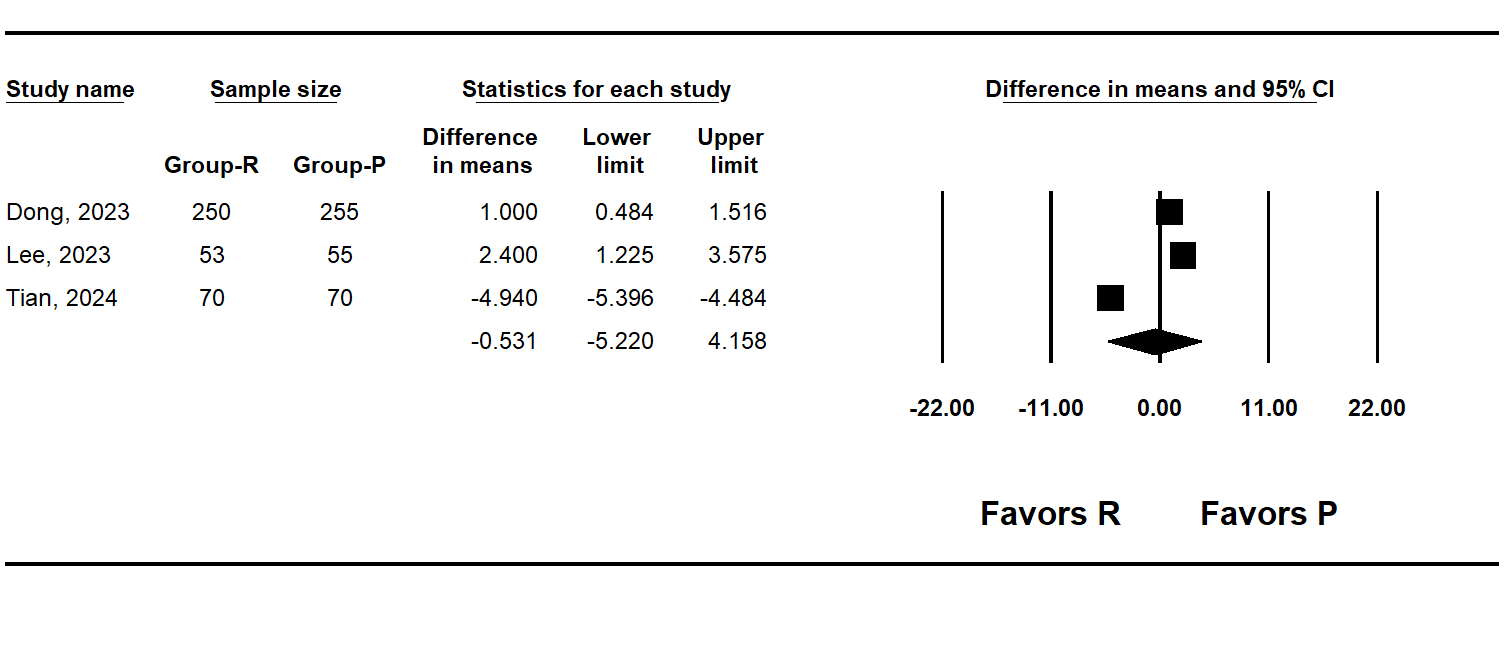


**Supplementary Figure S21. Trial sequential analysis plot for awake time comparing remimazolam and propofol.** Horizontal dotted red line represents the conventional boundaries for statistical significance. The blue solid line represents the cumulative z-curve. The TSA suggests insufficient evidence, with only 1.3% (753 of 57688 patients) of the required information size (RIS) accrued, as the Z-curve crossed neither the conventional test boundary nor cross the trial sequential monitoring boundary.


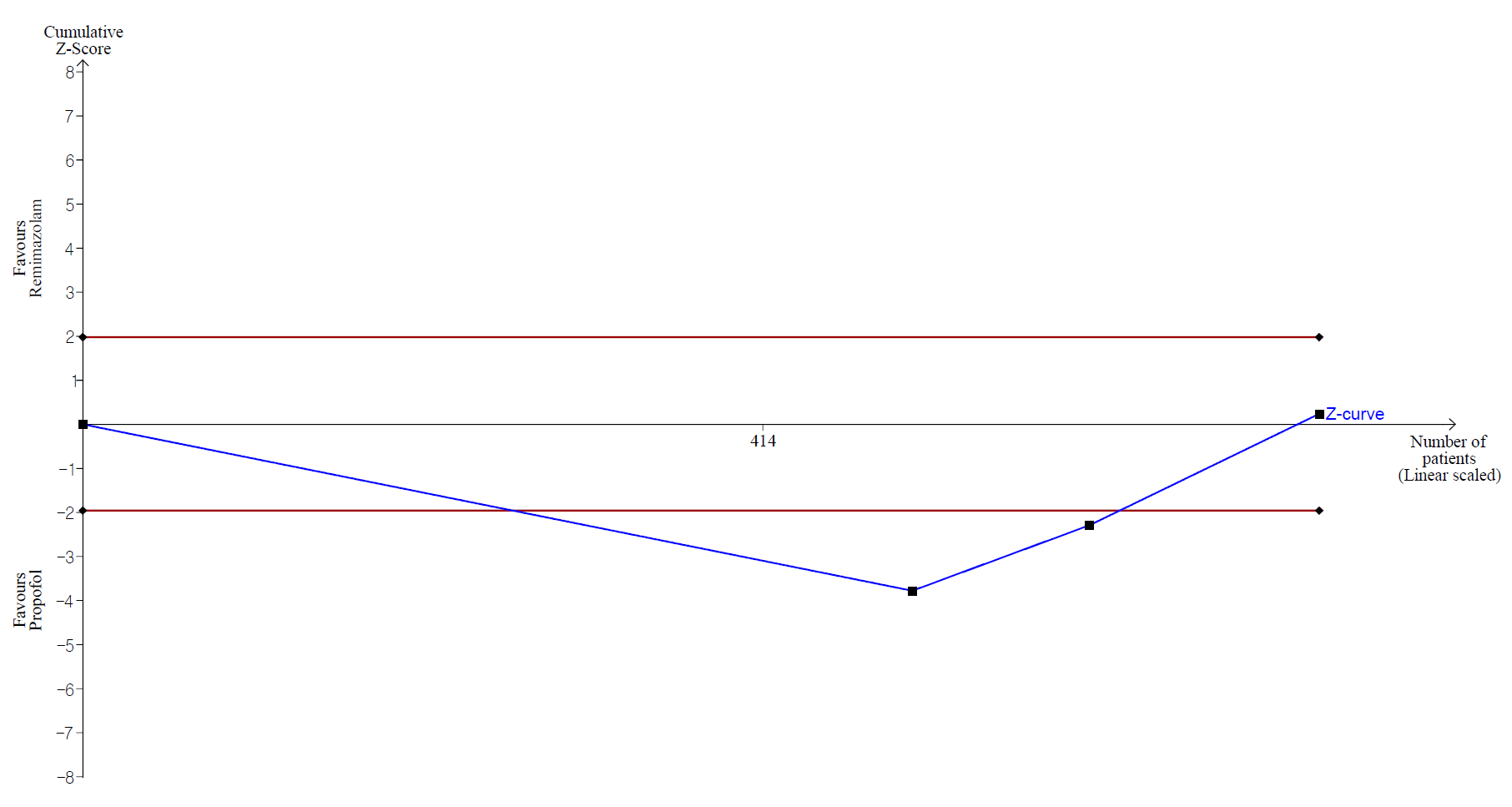


**Supplementary Figure S22. Forest plot for recovery time comparing remimazolam and propofol.** The figure depicts individual trials as filled squares with relative sample size and the 95% confidence interval (CI) of the difference as a solid line. The diamond shape indicates the pooled estimate and uncertainty for the combined effect. The pooled estimate indicates no significant difference in recovery time between remimazolam and propofol.


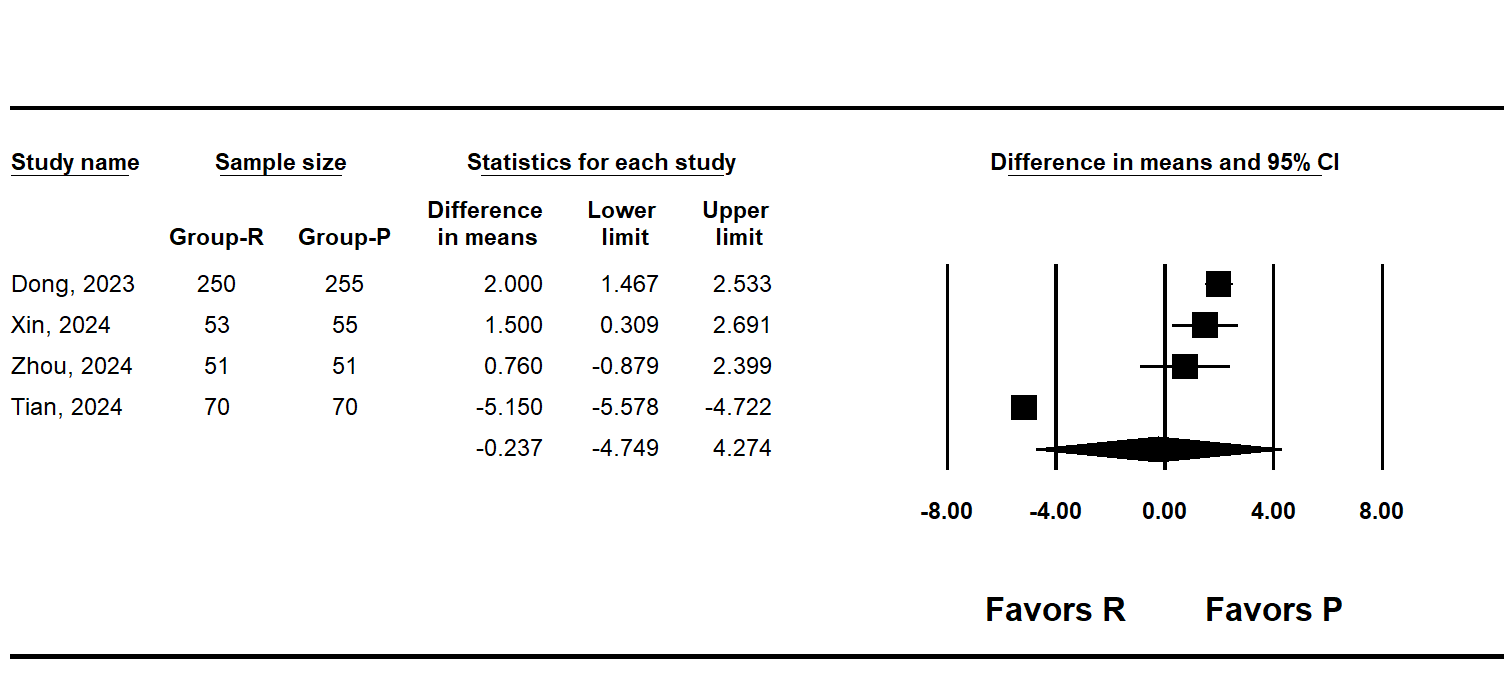


**Supplementary Figure S23. Trial sequential analysis plot for recovery time comparing remimazolam and propofol.** Horizontal dotted red line represents the conventional boundaries for statistical significance. The blue solid line represents the cumulative z-curve. The TSA suggests insufficient evidence, with only 1.5% (855 of 57892 patients) of the required information size (RIS) accrued, as the Z-curve crossed neither the conventional test boundary nor cross the trial sequential monitoring boundary.


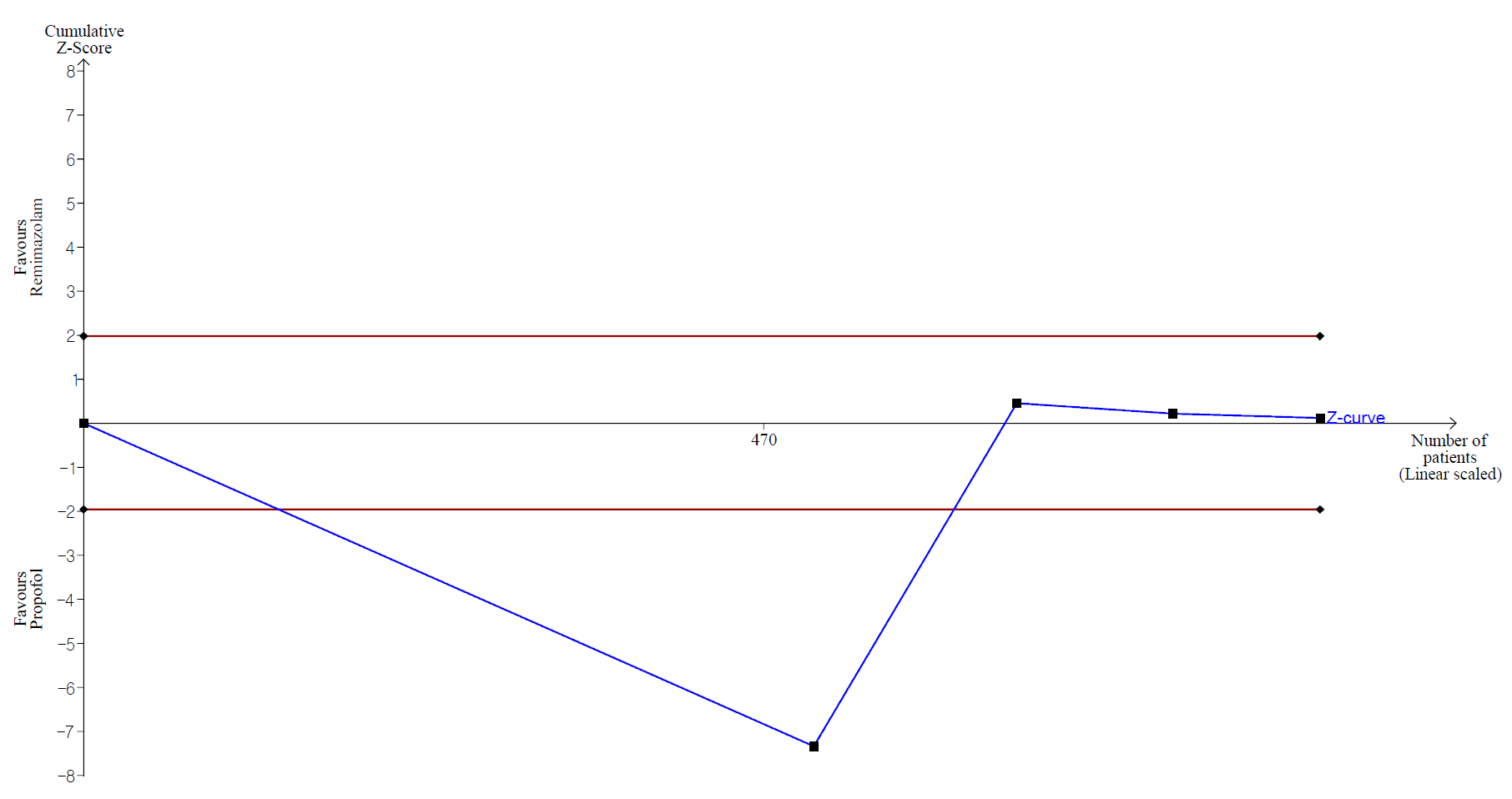


**Supplementary Figure S24. Forest plot for PACU time comparing remimazolam and propofol.** The figure depicts individual trials as filled squares with relative sample size and the 95% confidence interval (CI) of the difference as a solid line. The diamond shape indicates the pooled estimate and uncertainty for the combined effect. The pooled estimate indicates no significant difference in PACU time between remimazolam and propofol.


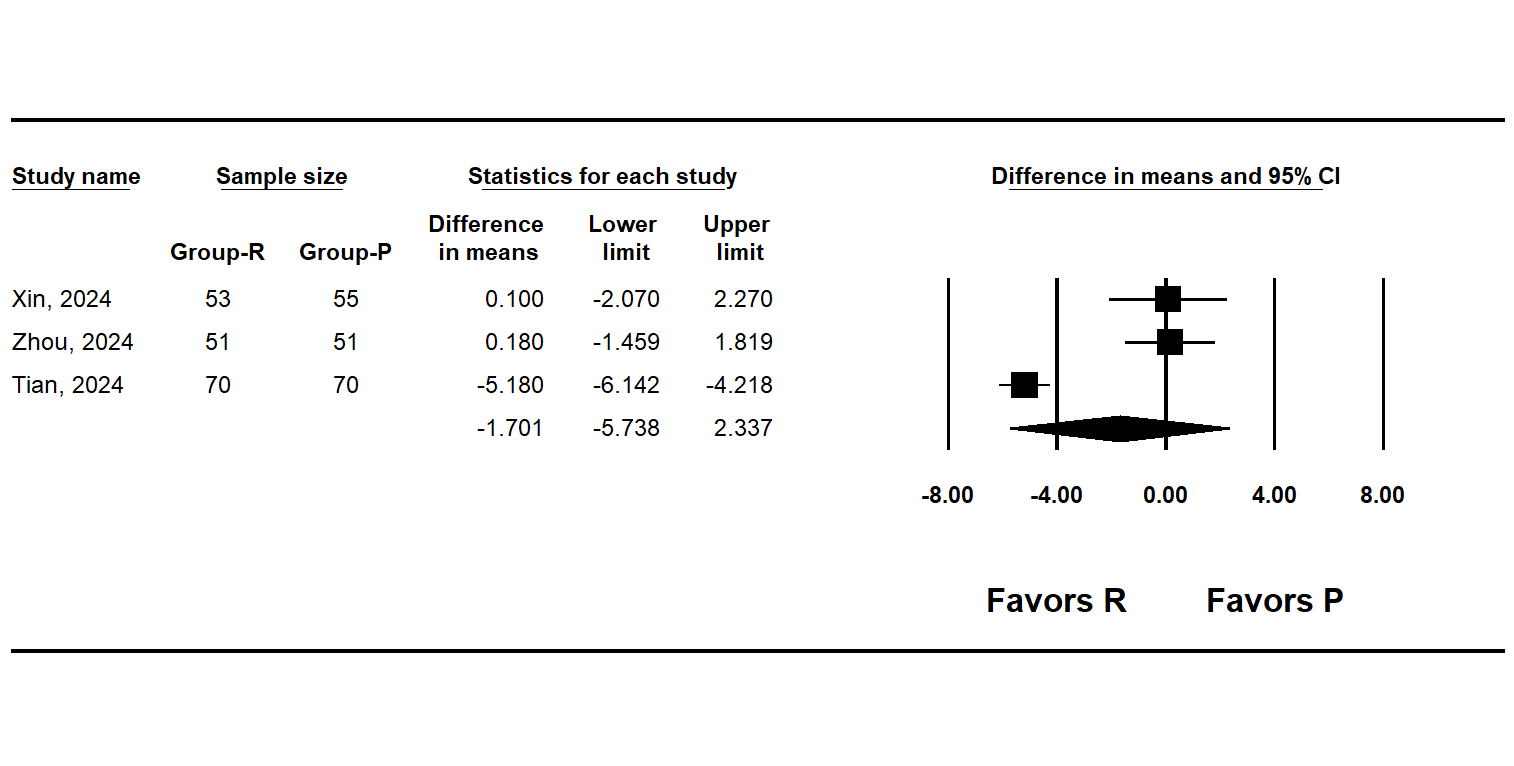


**Supplementary Figure S25. Trial sequential analysis plot for PACU time comparing remimazolam and propofol.** Horizontal dotted red line represents the conventional boundaries for statistical significance. The blue solid line represents the cumulative z-curve. The TSA suggests insufficient evidence, with only 4.56 % of the required information size (RIS) accrued, as the Z-curve crossed neither the conventional test boundary nor cross the trial sequential monitoring boundary.


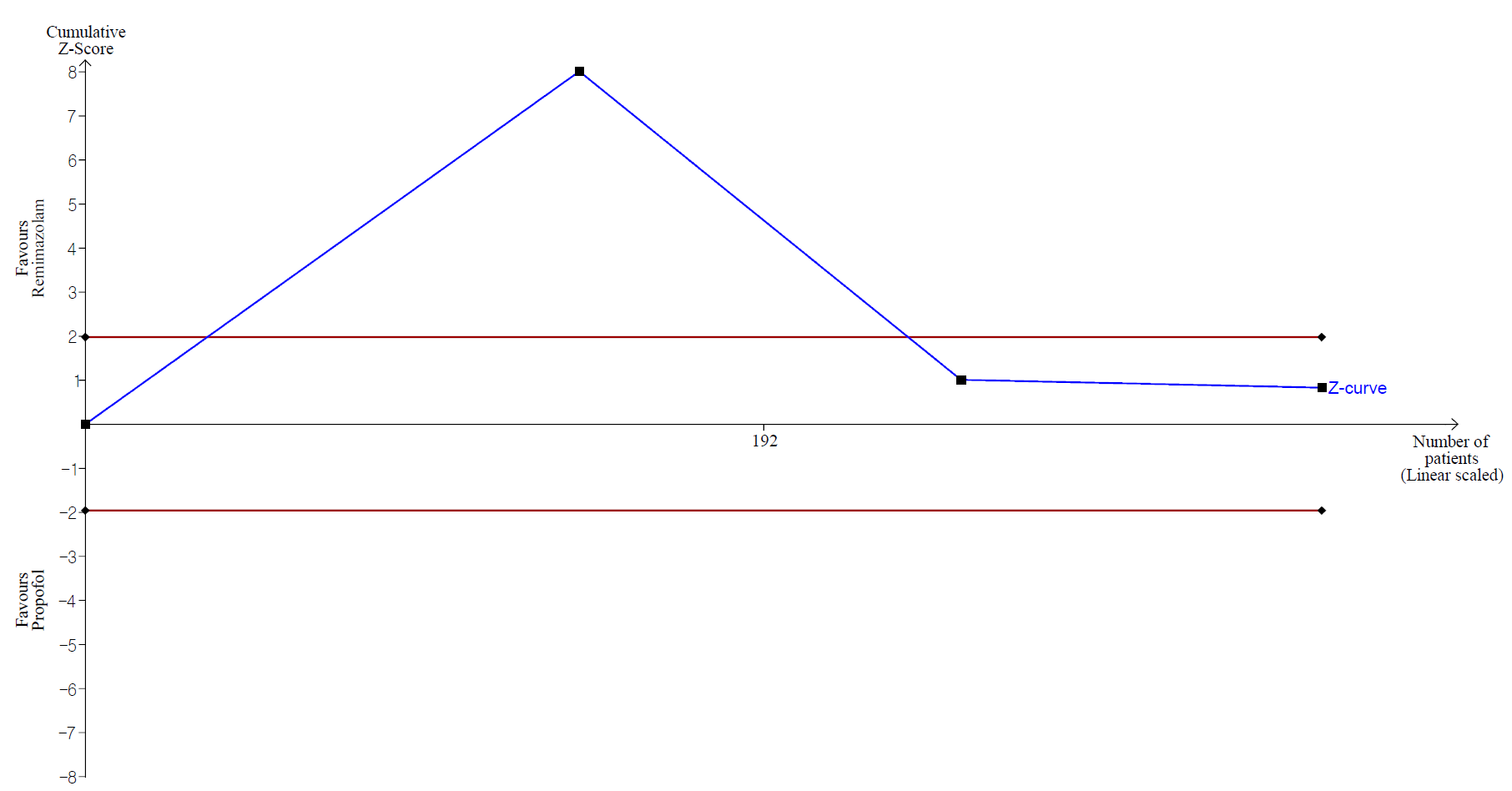


**Supplementary Figure S26. Forest plot for PONV comparing remimazolam and propofol.** The figure depicts individual trials as filled squares with relative sample size and the 95% confidence interval (CI) of the difference as a solid line. The diamond shape indicates the pooled estimate and uncertainty for the combined effect. The pooled estimate indicates no significant difference in PONV between remimazolam and propofol.


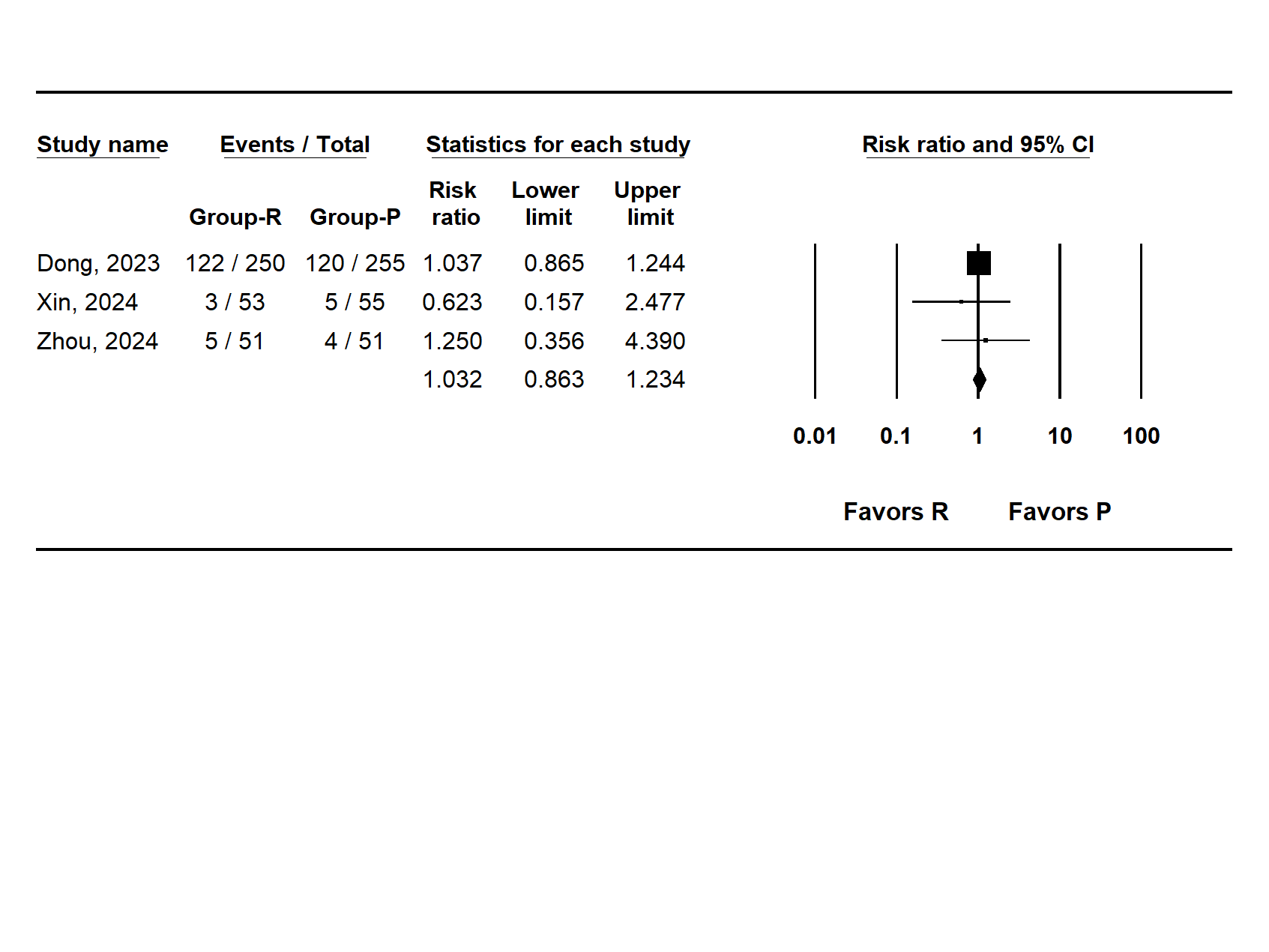


**Supplementary Figure S27. Trial sequential analysis plot for PONV comparing remimazolam and propofol.** Uppermost and lowermost complete red curves represent trial sequential monitoring boundary lines for benefit and harm respectively. Horizontal dotted red line represents the conventional boundaries for statistical significance. Triangular red lines on the right side reflects the futility boundaries. The blue solid line represents the cumulative z-curve. The number on the x-axis indicates required information size (n=579). The results show that patients enrolled exceeds RIS (735 of 446 patients), yet the cumulative Z curve intersects futility boundary, suggesting no further studies were needed.


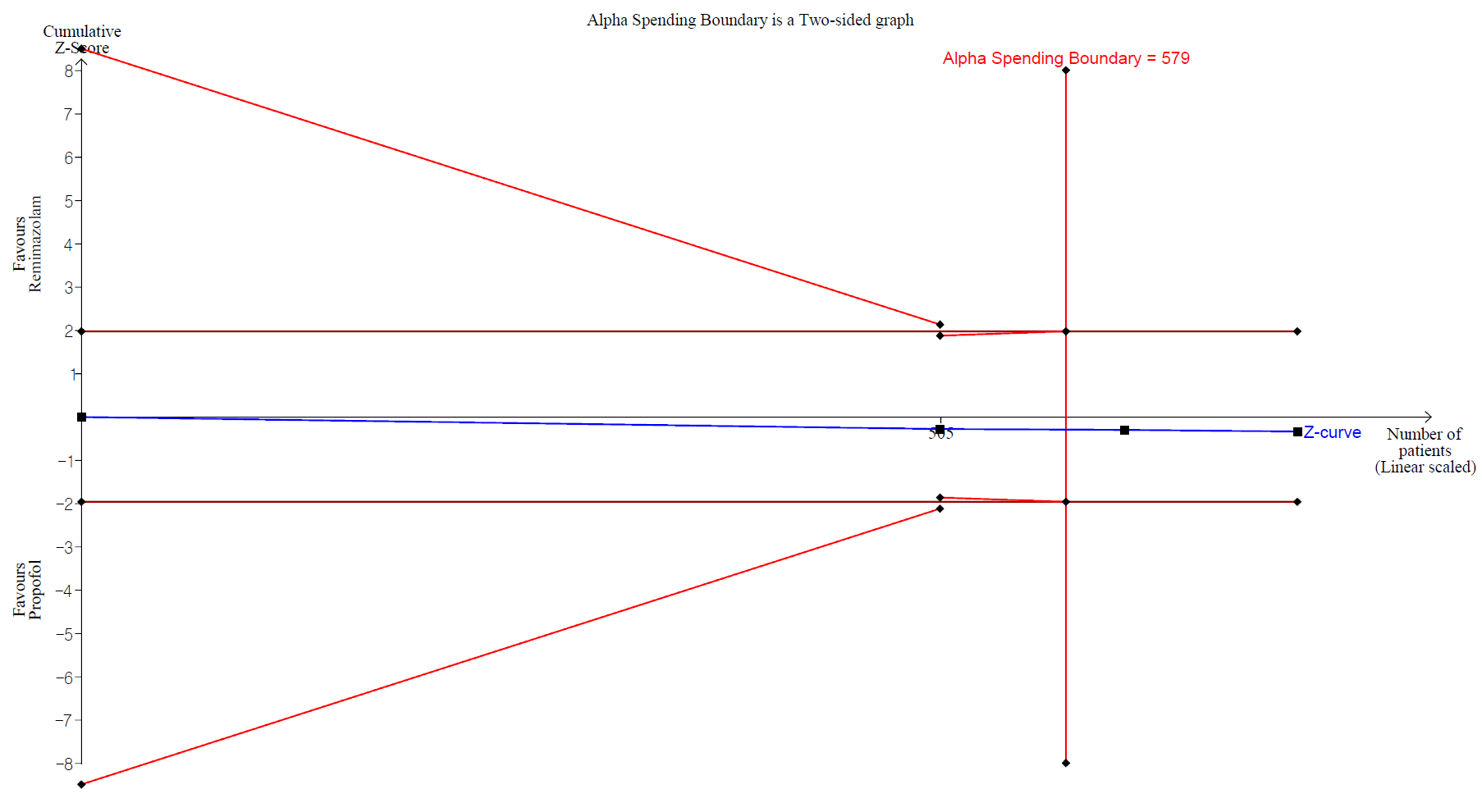


**Supplementary Figure S28. Forest plot for injection pain comparing remimazolam and propofol.** The figure depicts individual trials as filled squares with relative sample size and the 95% confidence interval (CI) of the difference as a solid line. The diamond shape indicates the pooled estimate and uncertainty for the combined effect. The pooled estimate indicates significant difference in injection pain between remimazolam and propofol.


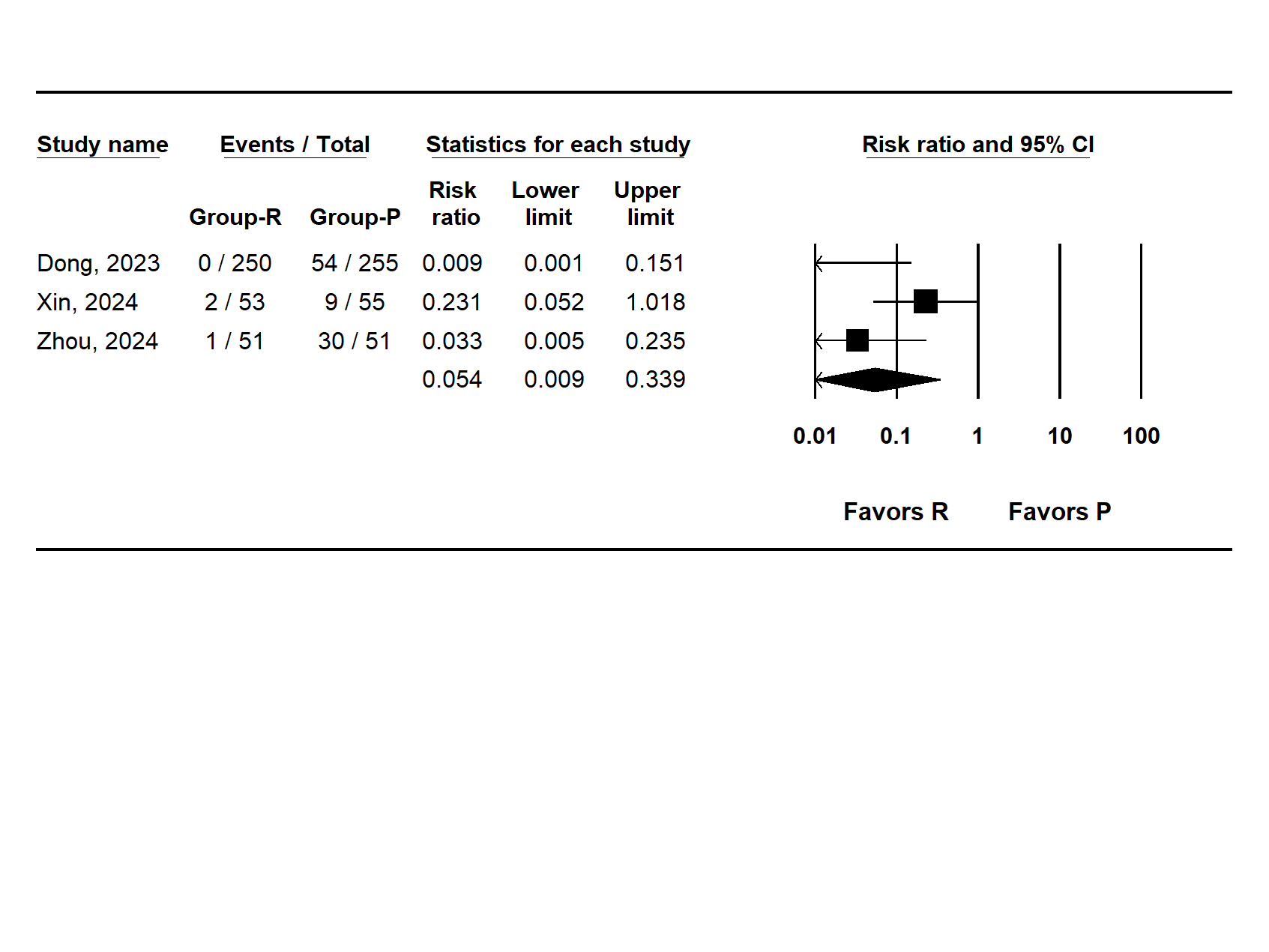


**Supplementary Figure S29. Trial sequential analysis plot for injection pain comparing remimazolam and propofol.** Uppermost and lowermost complete red curves represent trial sequential monitoring boundary lines for benefit and harm respectively. Horizontal dotted red line represents the conventional boundaries for statistical significance. Triangular red lines on the right side reflects the futility boundaries. The blue solid line represents the cumulative z-curve. The number on the x-axis indicates required information size (n=3410). The TSA suggests insufficient evidence, with only 21.0 % of the required information size (RIS) accrued, as the Z-curve crossed the conventional test boundary but did not cross the trial sequential monitoring boundary.


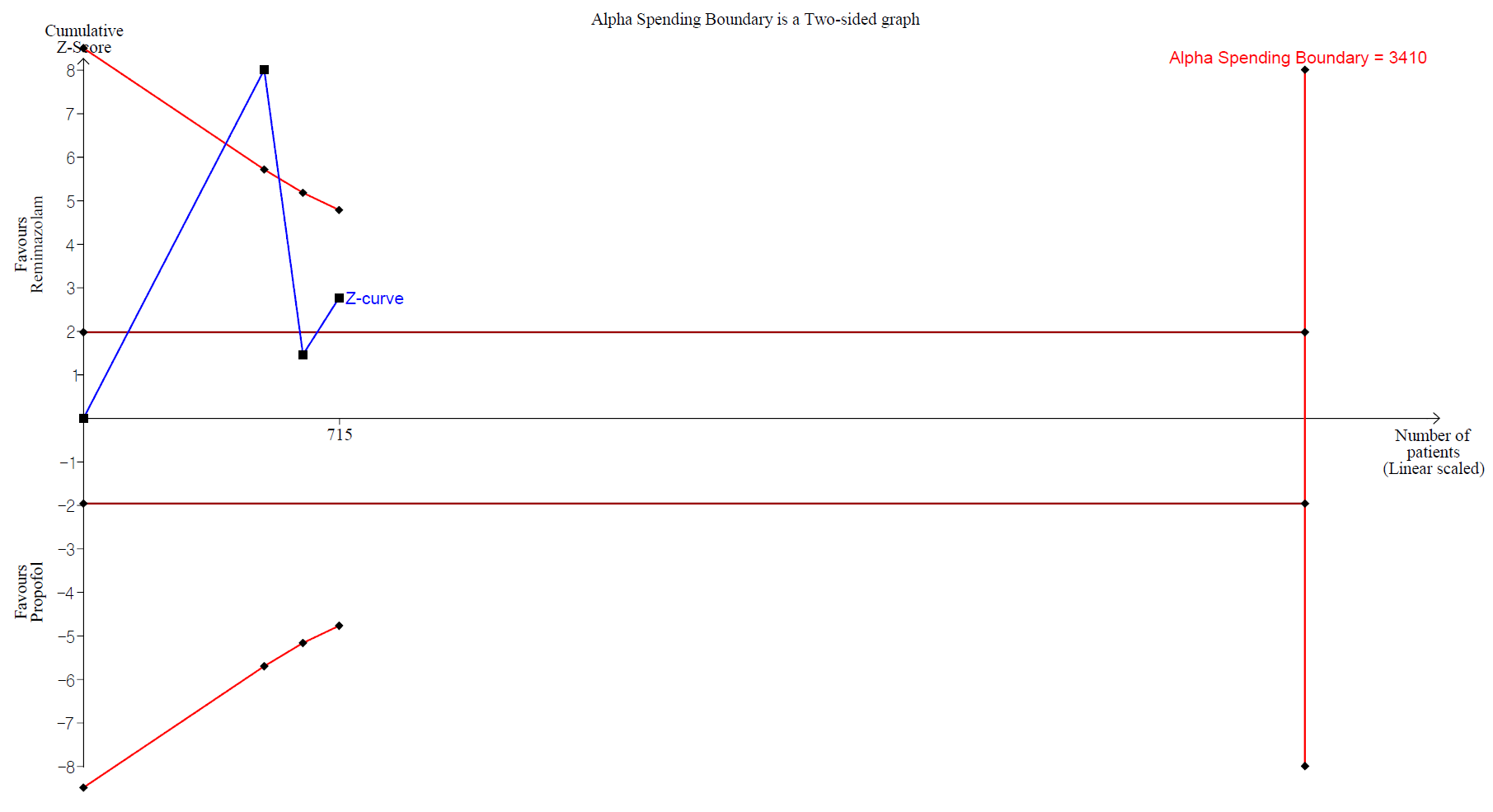


**Supplementary Table S1. The induction, maintenance and rescue doses of remimazolam, propofol and opioid**

| **Source** | **Opioid** | **Induction dose**  **(Remimazolam)** | **Maintenance dose** | **Rescue dose** | **Induction dose**  **(Propofol)** | **Maintenance dose** | **Rescue dose** |
| --- | --- | --- | --- | --- | --- | --- | --- |
| **Dong, 2023** | **alfentanil 10μg/kg before induction, maintenance 0-1 μg/kg/min** | **0.3mg/kg for >60 s** | **0.2-1 mg/kg/h** | **0.1mg/kg bolus & alfentanil 5μg/kg** | **1.5-2mg/kg propofol for 1 min** | **2-6 mg/kg/h** | **0.5mg/kg bolus & 5μg/kg alfentanil** |
| **Lee, 2023** |  | **5.0mg** |  | **2.5mg bolus upto 4 times (within 15 min. window, at least 2 min between doses)** | **0.5 mg/kg** |  | **0.5mg/kg bolus upto 4 times (within 15 min. window, at least 2 min between doses)** |
| **Xin, 2024** | **alfentanil 5.0μg/kg during induction, maintenance 0.5 μg/kg/min** | **0.15-0.20 mg/kg** | **maintenance 0.4-0.8 mg/kg/h** | **0.05mg/kg** | **1.0-1.5mg/kg** | **2.0-6.0 mg/kg/h** | **0.5mg/kg** |
| **Zhou, 2024** | **butorphanol tartrate 0.02mg/kg before induction, maintenance remifentanil 0.3μg/kg/h.** | **0.2–0.4mg/kg at a rate of 0.2–0.4 mg/kg/h** |  | **3–6 mg bolus & infusion pumping rate was increased by 10%** | **1–2.5mg/kg at a rate of 2–4 mg/kg/h** |  | **20–50 mg bolus & infusion pumping rate was increased by 10%** |
| **Tian, 2024** | **Sufentanil 5μg during induction, remifentanil during procedure (rate not specified)** | **0.2mg/kg for 1 min** |  | **2.5 mg bolus** | **1.5mg/kg** |  | **0.5mg/kg bolus** |

**Supplementary Table S2. Risk of bias**

| **Author, Year** | **Bias Arising from the Randomization Process** | **Bias Due to Deviations from Intended Intervention** | **Bias Due to Missing Outcome Data** | **Bias in Measurement of the Outcome** | **Bias in Selection of the Reported Results** | **Overall Bias** |
| --- | --- | --- | --- | --- | --- | --- |
| **Respiratory and Hemodynamic variables** | | | | | | |
| **Hypoxia** | | | | | | |
| Dong, 2023 | Low risk | Low risk^b^ | Low risk | Low risk | Low risk | Low risk |
| Lee, 2023 | Some concern ^a^ | Low risk^b^ | Low risk | Low risk^C^ | Low risk | Some concern |
| Xin, 2024 | Some concern ^a^ | Low risk^b^ | Low risk | Low risk | Low risk | Some concern |
| Zhou, 2024 | Low risk | Low risk^b^ | Low risk | Low risk^C^ | Low risk | Low risk |
| **Hypotension** | | | | | | |
| Dong, 2023 | Low risk | Low risk^b^ | Low risk | Low risk | Low risk | Low risk |
| Lee, 2023 | Some concern ^a^ | Low risk^b^ | Low risk | Low risk^C^ | Low risk | Some concern |
| Xin, 2024 | Some concern ^a^ | Low risk^b^ | Low risk | Low risk | Low risk | Some concern |
| Zhou, 2024 | Low risk | Low risk^b^ | Low risk | Low risk^C^ | Low risk | Low risk |
| **Hypertension** | | | | | | |
| Dong, 2023 | Low risk | Low risk^b^ | Low risk | Low risk | Low risk | Low risk |
| Lee, 2023 | Some concern ^a^ | Low risk^b^ | Low risk | Low risk^C^ | Low risk | Some concern |
| **Bradycardia** | | | | | | |
| Dong, 2023 | Low risk | Low risk^b^ | Low risk | Low risk | Low risk | Low risk |
| Lee, 2023 | Some concern ^a^ | Low risk^b^ | Low risk | Low risk^C^ | Low risk | Some concern |
| Xin, 2024 | Some concern ^a^ | Low risk^b^ | Low risk | Low risk | Low risk | Some concern |
| Zhou, 2024 | Low risk | Low risk^b^ | Low risk | Low risk^C^ | Low risk | Low risk |
| **Tachycardia** | | | | | | |
| Dong, 2023 | Low risk | Low risk^b^ | Low risk | Low risk | Low risk | Low risk |
| Lee, 2023 | Some concern ^a^ | Low risk^b^ | Low risk | Low risk^C^ | Low risk | Some concern |
| **Mean arterial pressure** | | | | | | |
| Dong, 2023 | Low risk | Low risk^b^ | Low risk | Low risk | Low risk | Low risk |
| Xin, 2024 | Some concern ^a^ | Low risk^b^ | Low risk | Low risk | Low risk | Some concern |
| Tian, 2024 | Some concern ^a^ | Low risk^b^ | Low risk | Low risk^C^ | Low risk | Some concern |
| **Heart rate** | | | | | | |
| Dong, 2023 | Low risk | Low risk^b^ | Low risk | Low risk | Low risk | Low risk |
| Xin, 2024 | Some concern ^a^ | Low risk^b^ | Low risk | Low risk | Low risk | Some concern |
| Tian, 2024 | Some concern ^a^ | Low risk^b^ | Low risk | Low risk^C^ | Low risk | Some concern |
| **Procedure condition** | | | | | | |
| **Completion rate** | | | | | | |
| Lee, 2023 | Some concern ^a^ | Low risk^b^ | Low risk | Low risk^C^ | Low risk | Some concern |
| Zhou, 2024 | Low risk | Low risk^b^ | Low risk | Low risk^C^ | Low risk | Low risk |
| **Procedure time** | | | | | | |
| Dong, 2023 | Low risk | Low risk^b^ | Low risk | Low risk | Low risk | Low risk |
| Lee, 2023 | Some concern ^a^ | Low risk^b^ | Low risk | Low risk^C^ | Low risk | Some concern |
| **Body movement** | | | | | | |
| Xin, 2024 | Some concern ^a^ | Low risk^b^ | Low risk | Low risk | Low risk | Some concern |
| Zhou, 2024 | Low risk | Low risk^b^ | Low risk | Some concern | Low risk | Some concern |
| **BIS** | | | | | | |
| Xin, 2024 | Some concern ^a^ | Low risk^b^ | Low risk | Low risk | Low risk | Some concern |
| Zhou, 2024 | Low risk | Low risk^b^ | Low risk | Low risk^C^ | Low risk | Low risk |
| Tian, 2024 | Some concern ^a^ | Low risk^b^ | Low risk | Low risk^C^ | Low risk | Some concern |
| **Times for sedation, procedure and recovery** | | | | | | |
| **Induction time** | | | | | | |
| Dong, 2023 | Low risk | Low risk^b^ | Low risk | Low risk | Low risk | Low risk |
| Lee, 2023 | Some concern ^a^ | Low risk^b^ | Low risk | Low risk^C^ | Low risk | Some concern |
| Zhou, 2024 | Low risk | Low risk^b^ | Low risk | Low risk^C^ | Low risk | Low risk |
| Tian, 2024 | Some concern ^a^ | Low risk^b^ | Low risk | Low risk^C^ | Low risk | Some concern |
| **Awake time** | | | | | | |
| Dong, 2023 | Low risk | Low risk^b^ | Low risk | Low risk | Low risk | Low risk |
| Lee, 2023 | Some concern ^a^ | Low risk^b^ | Low risk | Some concern^d^ | Low risk | High risk |
| Tian, 2024 | Some concern ^a^ | Low risk^b^ | Low risk | Some concern^d^ | Low risk | High risk |
| **Recovery time** | | | | | | |
| Dong, 2023 | Low risk | Low risk^b^ | Low risk | Low risk | Low risk | Low risk |
| Lee, 2023 | Some concern ^a^ | Low risk^b^ | Low risk | Some concern^d^ | Low risk | High risk |
| Xin, 2024 | Some concern ^a^ | Low risk^b^ | Low risk | Low risk | Low risk | Some concern |
| Zhou, 2024 | Low risk | Low risk^b^ | Low risk | Some concern^d^ | Low risk | Some concern |
| Tian, 2024 | Some concern ^a^ | Low risk^b^ | Low risk | Some concern^d^ | Low risk | High risk |
| **PACU time** | | | | | | |
| Xin, 2024 | Some concern ^a^ | Low risk^b^ | Low risk | Low risk | Low risk | Some concern |
| Zhou, 2024 | Low risk | Low risk^b^ | Low risk | Some concern^d^ | Low risk | Some concern |
| Tian, 2024 | Some concern ^a^ | Low risk^b^ | Low risk | Some concern^d^ | Low risk | High risk |
| **Other variables** | | | | | | |
| **Injection Pain** | | | | | | |
| Dong, 2023 | Low risk | Low risk^b^ | Low risk | Low risk | Low risk | Low risk |
| Xin, 2024 | Some concern ^a^ | Low risk^b^ | Low risk | Low risk | Low risk | Some concern |
| Zhou, 2024 | Low risk | Low risk^b^ | Low risk | Low risk^C^ | Low risk | Low risk |
| **Agitation and delirium** | | | | | | |
| Dong, 2023 | Low risk | Low risk^b^ | Low risk | Low risk | Low risk | Low risk |
| Xin, 2024 | Some concern ^a^ | Low risk^b^ | Low risk | Low risk | Low risk | Some concern |
| Zhou, 2024 | Low risk | Low risk^b^ | Low risk | Low risk^C^ | Low risk | Low risk |
| **PONV** | | | | | | |
| Dong, 2023 | Low risk | Low risk^b^ | Low risk | Low risk | Low risk | Low risk |
| Xin, 2024 | Some concern ^a^ | Low risk^b^ | Low risk | Low risk | Low risk | Some concern |
| Zhou, 2024 | Low risk | Low risk^b^ | Low risk | Some concern^d^ | Low risk | Some concern |
| **Quality of recovery at postoperative day 1** | | | | | | |
| Dong, 2023 | Low risk | Low risk^b^ | Low risk | Low risk | Low risk | Low risk |
| Xin, 2024 | Some concern ^a^ | Low risk^b^ | Low risk | Low risk | Low risk | Some concern |

^a^ No information on the allocation concealment

^b^ Personnel aware of intervention, but no deviation from intended intervention.

^c^ No information on blinding of outcome assessor but outcome assessment was not influenced by knowledge of intervention

^d^. No information on blinding of outcome assessor and outcome assessment was influenced by knowledge of intervention

**Supplementary Table S3. The GRADE evidence quality for each outcome**

|  | No of studies | Quality assessment | | | | | Quality |
| --- | --- | --- | --- | --- | --- | --- | --- |
|  |  | ROB | Inconsistency | Indirectness | Imprecision | Publication bias |  |
| **Hypoxia** | 4 | not serious | not serious | not serious | not serious | NA | ⨁⨁⨁⨁ High |
| **Hypotension** | 4 | not serious | not serious | not serious | not serious | NA | ⨁⨁⨁⨁ High |
| **Hypertension** | 2 | not serious | not serious | not serious | serious^b^ | NA | ⨁⨁⨁◯ Moderate |
| **Bradycardia** | 4 | not serious | not serious | not serious | not serious | NA | ⨁⨁⨁⨁ High |
| **Tachycardia** | 2 | not serious | not serious | not serious | serious^b^ | NA | ⨁⨁⨁◯ Moderate |
| **Mean arterial pressure** | 3 | not serious | serious^a^ | not serious | serious^b^ | NA | ⨁⨁◯◯ Low |
| **Heart rate** | 3 | not serious | not serious | not serious | serious^b^ | NA | ⨁⨁⨁◯ Moderate |
| **Completion rate** | 2 | not serious | not serious | not serious | serious^b^ | NA | ⨁⨁⨁◯ Moderate |
| **Procedure time** | 2 | not serious | serious^a^ | not serious | serious^b^ | NA | ⨁⨁◯◯ Low |
| **Body movement** | 2 | not serious | not serious | not serious | serious^b^ | NA | ⨁⨁⨁◯ Moderate |
| **BIS** | 3 | not serious | serious^a^ | not serious | serious^b^ | NA | ⨁⨁◯◯ Low |
| **Induction time** | 4 | not serious | serious^a^ | not serious | serious^b^ | NA | ⨁⨁◯◯ Low |
| **Awake time** | 3 | not serious | serious^a^ | not serious | serious^b^ | NA | ⨁⨁◯◯ Low |
| **Recovery time** | 4 | not serious | serious^a^ | not serious | serious^b^ | NA | ⨁⨁◯◯ Low |
| **PACU time** | 3 | not serious | serious^a^ | not serious | serious^b^ | NA | ⨁⨁◯◯ Low |
| **Injection Pain** | 3 | not serious | serious^a^ | not serious | serious^b^ | NA | ⨁⨁◯◯ Low |
| **Agitation and delirium** | 3 | not serious | not serious | not serious | serious^b^ | NA | ⨁⨁⨁◯ Moderate |
| **PONV** | 3 | not serious | not serious | not serious | serious^b^ | NA | ⨁⨁⨁◯ Moderate |
| **POD1_QOR15** | 2 | not serious | serious^a^ | not serious | serious^b^ | NA | ⨁⨁◯◯ Low |

No.; number, NA; not assessed, BIS; Bispectral Index, PACU; post-anesthesia care unit, PONV; post-operative nausea and vomiting, POD1_QOR15; 15-item questionnaire on quality of recovery at postoperative day 1.

GRADE Working Group grades of evidence
High certainty: we are very confident that the true effect lies close to that of the estimate of the effect.
Moderate certainty: we are moderately confident in the effect estimate: the true effect is likely to be close to the estimate of the effect, but there is a possibility that it is substantially different.
Low certainty: our confidence in the effect estimate is limited: the true effect may be substantially different from the estimate of the effect.
Very low certainty: we have very little confidence in the effect estimate: the true effect is likely to be substantially different from the estimate of effect.

^a^ We downgraded the certainty of evidence by one level for inconsistency due to heterogeneity among studies, with an I^2^ statistic over 50% or P_chi_^2^ less than 0.05.

^b^ We downgraded imprecision by one levels for serious imprecision due to very wide confidence intervals, including both substantial harms and benefits.
